# Stochastic loss and gain of symmetric divisions in the *C*. *elegans* epidermis perturbs robustness of stem cell number

**DOI:** 10.1371/journal.pbio.2002429

**Published:** 2017-11-06

**Authors:** Dimitris Katsanos, Sneha L. Koneru, Lamia Mestek Boukhibar, Nicola Gritti, Ritobrata Ghose, Peter J. Appleford, Maria Doitsidou, Alison Woollard, Jeroen S. van Zon, Richard J. Poole, Michalis Barkoulas

**Affiliations:** 1 Department of Life Sciences, Imperial College, London, United Kingdom; 2 Institute for Atomic and Molecular Physics (AMOLF), Amsterdam, The Netherlands; 3 Department of Biochemistry, University of Oxford, Oxford, United Kingdom; 4 Centre for Integrative Physiology, University of Edinburgh, Edinburgh, United Kingdom; 5 Department of Cell and Developmental Biology, University College London, London, United Kingdom; New York University, United States of America

## Abstract

Biological systems are subject to inherent stochasticity. Nevertheless, development is remarkably robust, ensuring the consistency of key phenotypic traits such as correct cell numbers in a certain tissue. It is currently unclear which genes modulate phenotypic variability, what their relationship is to core components of developmental gene networks, and what is the developmental basis of variable phenotypes. Here, we start addressing these questions using the robust number of *Caenorhabditis elegans* epidermal stem cells, known as seam cells, as a readout. We employ genetics, cell lineage tracing, and single molecule imaging to show that mutations in *lin-22*, a *Hes*-related basic helix-loop-helix (bHLH) transcription factor, increase seam cell number variability. We show that the increase in phenotypic variability is due to stochastic conversion of normally symmetric cell divisions to asymmetric and vice versa during development, which affect the terminal seam cell number in opposing directions. We demonstrate that LIN-22 acts within the epidermal gene network to antagonise the Wnt signalling pathway. However, *lin-22* mutants exhibit cell-to-cell variability in Wnt pathway activation, which correlates with and may drive phenotypic variability. Our study demonstrates the feasibility to study phenotypic trait variance in tractable model organisms using unbiased mutagenesis screens.

## Author summary

Organisms are exposed to both internal and external perturbations in every molecular process they go through, and robustness—the ability to maintain their systems unchanged—is crucial for their development and survival. However, the processes that keep the variability of cells as low as possible are barely known. The nematode *C*. *elegans* is notable for its highly reproducible development, showing an almost invariant pattern of cell division and differentiation during development; it is thus an ideal model organism in which to search for genes that regulate phenotypic consistency among genetically identical individuals. We focus on a group of lateral epidermal cells—the seam cells—which undergo stem cell-like divisions during postembryonic development. These divisions can either be symmetric towards the seam cell fate, acting to increase the total number of cells, or asymmetric, giving rise to one daughter cell that differentiates into its final fate and another one that serves to keep the number of seam cells constant. We show here that mutations in the transcription factor *lin-22* increase seam cell number variability due to stochastic conversion of symmetric divisions into asymmetric ones and vice versa during development, thereby altering the number of terminal seam cell number in opposing directions. We also show that the observed phenotypic variability correlates with the stochastic activation of the conserved Wnt signaling pathway. Our work suggests that core components of developmental gene networks modulate phenotypic variability in multicellular animals.

## Introduction

It is remarkable how biological systems manage to operate consistently despite facing several types of variation, including the intrinsic stochasticity in every molecular process. This ability of a given system to produce an invariable output in the presence of internal and external perturbations is called robustness [1, 2]. Developmental processes need to be robust to perturbations to achieve balanced growth and morphogenesis. This includes stem cell number regulation, which protects an organism from tissue hyperplasia, while at the same time facilitates tissue maintenance and repair. Recent advances in gene expression and protein quantification with single-cell resolution have suggested a substantial amount of cell-to-cell molecular heterogeneity in biological systems [3], raising the question of how robustness is achieved at the phenotypic level.

Since Waddington, who first discussed developmental variability [4], there is a growing interest in understanding phenotypic buffering using both theoretical and experimental approaches [5]. To this end, a key goal is to discover which genes influence phenotypic variance (Box 1). Although it has previously been shown that disruption of single genes can lead to phenotypic variability, most experimental studies have been targeted to unicellular organisms or tested specific candidates, usually heat-shock proteins [6–9]. For example, the chaperone HSP90 is often thought to play a major buffering role by suppressing phenotypic variability in animals and plants, thereby allowing genetic variation to accumulate in a cryptic form [9, 10]. However, the developmental mechanisms underlying variability upon *Hsp90* impairment are not understood. Furthermore, recent evidence has suggested a more complex picture as perturbations of *Hsp90* can also reduce phenotypic variance, indicating a dual role for this chaperone as either a potentiator of variability or buffer [11]. To date, genome-wide mutagenesis screens to identify factors shaping phenotypic variability have not been performed in multicellular animals. Therefore, it remains largely unclear: (1) what are the genes that modulate developmental trait variance as a response to a specific perturbation, (2) how these genes fit in developmental gene networks, and (3) what their specificity is to the phenotypic trait of interest within the context of a whole organism.

**Box 1. Developmental robustness and variance–modulating genes.**

Robustness is the ability of a biological system to withstand perturbations, such as changes in the environment or the inherent stochasticity of molecular processes (noise), in order to produce a consistent output [2, 5, 21]. By extending this definition to a phenotypic trait and development, a robust phenotype is characterised by lack of, or low, variation under a given perturbation. Developmental phenotypes can be studied at many different levels of biological organisation, therefore robust and variable phenotypes most often coexist within a given system. For example, phenotypes can range from morphology to behaviour or signalling pathway activity to gene expression. When considering multicellular organisms, phenotypic variation can be quantified among related cells within a single individual reflecting cell-to-cell heterogeneity or among different individuals of the population.

Mutations in certain genes can lead to loss of developmental robustness resulting in a significant increase in phenotypic variability. To study phenotypic variability as a consequence of reduced robustness, one needs to consider the phenotypic distribution of the population, as opposed to simply relying on phenotypic averages [22]. When quantifying phenotypic variability, one concern is that many developmental mutants are phenotypically more variable than the wild-type (WT); therefore, an increase in variance may be a by-product of differences in the mean [4]. One way to address this is by specifically studying mutations that cause changes in variance, selecting against those that change the mean [2]. Another possibility is to take into account the complex relationship between variance and mean for each phenotype and study cases in which a change in variance is higher than expected for a given value of the mean [6]. In cases in which phenotypic variance increases while the mean is the same, the phenotypic distribution expands on both sides of the mean, leading to 2-sided phenotypic variants. These are interesting to study developmentally and might be useful as a proxy for distinguishing from mutations with low penetrance, which might also increase variance, but without showing 2-sided phenotypic effects [2]. However, developmental constraints may allow phenotypic variants on one side of the distribution only.

This study focuses on mutations inducing seam cell number variability to noise among isogenic *C*. *elegans* individuals in the population. We refer here to genes that, when mutated or deleted, lead to an increase in phenotypic variance to a given perturbation as variance-modulating genes (also called phenotypic capacitors in the literature). Discovering such genes and characterising their mode of action is a fundamental problem in biology that relates to the genotype-to-phenotype mapping. Variance-modulating genes can be identified through various experimental methods. Genetic screens focusing on trait variance as the phenotype of interest have not been previously attempted in *C*. *elegans*, but have been performed in yeast using quantitative morphology or reporter gene expression as a read-out [6, 8, 23]. More targeted genetic efforts in flies have contributed to identifying genomic regions or single genes acting to minimise developmental variability [24–26]. Quantitative trait loci (QTL) approaches and genome-wide association studies (GWAS) have also been useful to identify loci controlling trait variance in natural populations [7, 27–32]. Variance-modulating genes in yeast have been linked to pleiotropy, influencing variance of many independent phenotypes [23]. Developmental variability across phenotypes may also arise due to sickness or decrease in organismal fitness, which can be addressed by quantifying certain fitness traits [23, 33]. Variance-modulating factors may have multiple molecular gene functions and although they can influence trait variability, they may not necessarily evolve due to selection for phenotypic robustness [2].

A series of questions with regard to genes influencing developmental variability in multicellular animals remain to be answered. For instance, it is not well understood what the difference is between genes affecting trait mean and variance and how variance-modulating genes fit in core developmental gene networks. Moreover, the specificity of these genes to a phenotype or perturbation of interest is underexplored, thus it is unknown how common system-wide effects are. Most importantly, the developmental basis of phenotypic variability even for well-documented cases, such as upon *Hsp90* impairment, has not been explored.

Here, we address these questions using *C*. *elegans* as a model. Developmental patterning in *C*. *elegans* is highly stereotypical and these animals are thought to be near-eutelic; that is, there is an almost invariant number of 959 somatic cells present in every adult hermaphrodite [12]. Furthermore, the complete lineage of all cells is known, allowing precise tracing of developmental defects with single-cell resolution [13]. Importantly, *C*. *elegans* populations are also isogenic due to their hermaphroditic reproductive mode. This eliminates a key confounder when studying phenotypic variance in a population, which is the presence of standing genetic variation.

**Fig 1:**
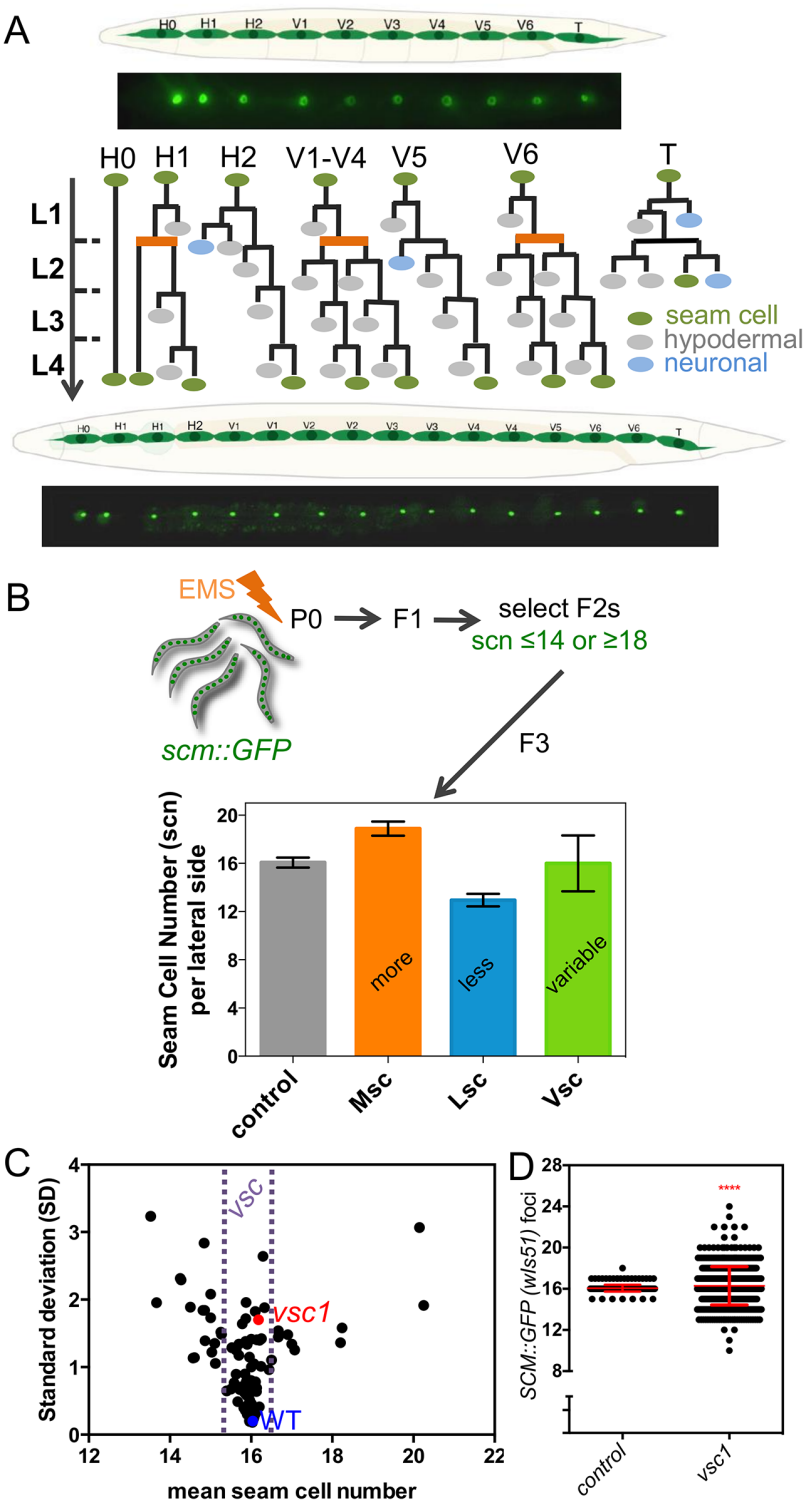
Recovery and mapping of mutants with variable seam cell number. (A) Cartoon illustrating the seam cell lineages in WT. Fluorescent images show expression of 10 *scm*∷*GFP* (*wIs51*) positive cells at the L1 (above) and 16 cells at the early adult stage (below). (B) Design of the genetic screen to recover mutants with a Vsc phenotype as opposed to Msc or Lsc, based on selection of extreme seam cell number at the F2 generation. Control represents representative data for JR667 (*wIs51*) strain, black bar shows mean ± SD. (C) Relationship between SD and mean scn. Each point represents an independently recovered mutant from our EMS screen. Control strain JR667 is depicted in blue and the *vsc1* mutant in red. (D) *vsc1* mutants show variable seam cell numbers (SD = 0.33, *n* = 278 animals for control JR667, and SD = 1.87, *n* = 563 for *vsc1* mutants). Note that only 1 animal shows extreme seam cell counts in this experiment in WT. Error bar shows mean ± SD and red stars depict statistically significant change in variance in relationship to control with a Levene’s median test (*P* < 0.0001). Numerical data used for Fig 1, B, C, D can be found in S2 Data. SD, standard deviation; EMS, ethyl methanesulfonate; GFP, green fluorescent protein; L1, first larval stage; Vsc, variable seam cell number phenotype; Lsc, less seam cells phenotype; Msc, more seam cells phenotype; SCM, seam cell marker; scn, seam cell number; WT, wild-type.

We particularly focus on seam cells, which are found on both lateral sides of the nematode and contribute to cuticle secretion together with the surrounding hypodermis [14]. The seam cells show stem cell-like properties dividing in a symmetric or asymmetric manner during postembryonic development (Fig 1A). More precisely, animals hatch with 10 embryonically born seam cells per lateral side, and that number increases to 16 after the early second larval stage (L2) due to a symmetric division. The cells also pass through a series of reiterative asymmetric cell divisions during all larval stages, after which one daughter cell differentiates into a neuronal precursor cell or fuses with the syncytial hypodermis, while the (usually) posterior daughter cell maintains the stem cell potential (Fig 1A).

The postembryonic lineage behaviour of seam cells is not uniform along the anterior-posterior axis. Cell division patterns differ, for example, between the head seam cells (H0–H2) and the seam cells in the mid body (V1–V6) or tail (T), and also within these groups of cells. Seam cell development has been shown to be influenced by a combination of transcription factor activities including GATA factors and the RNT-1/BRO-1 (Runx1/CBFβ) module [15–18], conserved signaling pathways such as the Wnt pathway [19], and the heterochronic gene pathway that regulates developmental timing in *C*. *elegans* [20].

Seam cell number in the widely used laboratory reference strain N2 is consistent with a WT mean of 16 cells per lateral side in the early adult. However, this phenotype is not fully invariant, as it is evident by the low penetrance of animals in the population (typically around 10%) showing either more or fewer seam cells (mostly 17 and 15 cells, respectively). To explore mechanisms of developmental robustness, we initiated in this study a forward genetic approach to identify mutants showing a significant increase in the variability of terminal seam cell number, indicative of animal-to-animal variability within the population. We demonstrate that mutations in *lin-22*, a Hairy/Enhancer of Split (*Hes)*-related bHLH transcription factor, increase seam cell number variance via stochastic loss and gain of symmetric divisions that occur within single animals and occasionally within the same epidermal lineage. Loss of symmetric divisions at L2 give rise to more neuroblasts at the expense of seam cells, while symmetric divisions towards the seam cell fate at subsequent developmental stages increase the seam cell pool. We show that *lin-22* is a core component of the seam cell developmental gene network interacting with the Wnt signaling pathway so that *lin-22* null mutants show stochastic Wnt pathway activation that correlates with phenotypic variability. We finally study systemic effects in the nematode and show that gain in variability in seam cell patterning is accompanied by loss of stochasticity or no change in other developmental contexts.

## Results

### A genetic screen for modulators of seam cell number variance identifies *vsc1*

To study the genetic mechanisms underpinning the consistency of seam cell number among individuals, we set out to isolate mutants showing an increase in seam cell number variance. To this end, we mutagenised a strain harbouring an integrated *scm*∷*GFP* transgene (*wIs51*) that is commonly used as a seam cell marker [15,34], allowed the F1 animals to produce self-progeny and selected F2 individuals showing an “extreme” seam cell number phenotype, as defined by a seam cell count that is either lesser than 15 or greater than 17 cells per lateral side. This extreme phenotype is very rare (<1%) among WT animals. Variability is defined in this screen at the level of the population, so we hypothesised that the selected animals would either show in the next generation a variable seam cell number phenotype (Vsc) or alternatively an increase (more seam cells phenotype [Msc]) or decrease (less seam cells phenotype [Lsc]) in terminal seam cell number (Fig 1B). Changes in mean and variance are not mutually exclusive and the relationship between these 2 measures depends on the developmental system of choice [2]. We found that the seam cell number variability increases when the phenotypic mean departs in any direction (increase or decrease) from the average of 16 cells per lateral side. Therefore, to be confident about a variance change we decided to focus on variable mutants (*vsc*) in which the phenotypic mean showed only minimal change compared to the WT (Fig 1C). We also concentrated on mutants showing 2-sided errors—that is, at the same time both an increase and a decrease in seam cell number within the isogenic population—in an attempt to dissect the developmental basis of bidirectional variability (Box 1).

One of the recovered mutants that satisfied the above criteria was *vsc1*. This mutant showed a statistically significant increase in seam cell number variance without a drastic change in the mean (WT = 16.05 ± 0.33 SD versus *vsc1* = 16.29 ± 1.87 SD) and phenotypic errors on both sides of the mean (Fig 1D). Interestingly, *vsc1*, like other recovered *vsc* mutants, showed higher seam cell number variability compared to that observed upon impairment of the expression of genes often considered as bona fide buffering factors such as *Hsp90*/*daf-21* (SD = 0.93; S1A Fig). We therefore sought to identify these variance modulators and aimed at mapping the molecular lesion in *vsc1*. A current method to identify causative mutations from mutagenesis experiments in *C*. *elegans* relies on “mapping-by-sequencing” after crossing to the polymorphic *C*. *elegans* isolate CB4856 [35,36]. We selected homozygous F2 recombinants based on phenotypic similarity of their progeny to the mutant parental strain, relying on a metric of phenotypic variability (SD), the percentage of extremes in the population and the percentage of animals showing 16 seam cells (S1B Fig). After pooling together these lines and whole genome sequencing, we identified a region of around 0.5–1 Mb on the left arm of chromosome IV that contained exclusively N2 markers and was thus likely to harbour the causative mutation for seam cell number variability (S1C Fig).

### The causative mutation in *vsc1* maps upstream of the transcription factor *lin-22*

**Fig 2:**
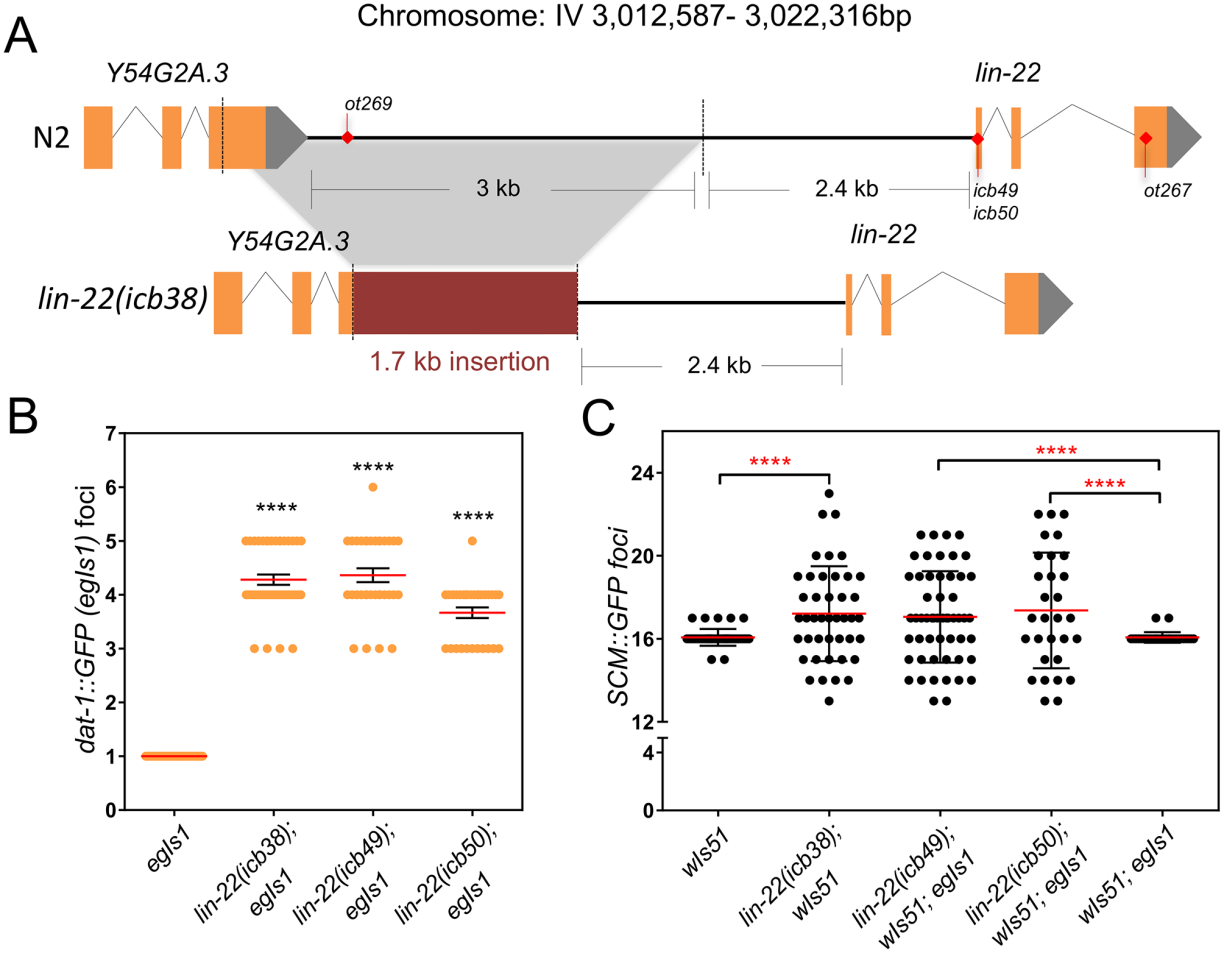
The *icb38* mutation represents a loss of function mutation in *lin-22*. (A) Illustration of the *lin-22(icb38)* mutation, which is a 3,329 bp deletion removing part of the distal *lin-22* promoter (2,371 bp upstream of the *lin-22* ATG). The deletion also removes part of the third exon and 3′ UTR of the upstream gene Y54G2A.3. The deleted part is replaced by a 1,733 bp insertion consisting of exon 7 and parts of introns 6 and 7 of the downstream gene *mca-3*. The position of other *lin-22* alleles described in the manuscript is shown on the wild-type sequence. (B) Quantification of the number of PDE neurons (*dat-1*∷*GFP* foci) in the EMS-derived *lin-22(icb-38)* mutant and CRISPR-derived *lin-22* mutants (*n* ≥ 30). Reference sample is *egIs1* containing only the marker. (C) Quantification of seam cell number in *lin-22(icb38)* and other CRISPR-derived *lin-22* mutants (*n* ≥ 30). Note an increase in seam cell number variance in *lin-22* mutants depicted with red stars. Black stars show statistically significant changes in the mean with one-way ANOVA followed by the Dunnett test, and red stars depict changes in variance with a Levene’s median test (in both cases, **** corresponds to *P* value < 0.0001). Error bars show mean ± SEM (B) or mean ± SD (C). Numerical data used for Fig 2B, C can be found in S2 Data. CRISPR, Clustered Regularly Interspaced Short Palindromic Repeats; EMS, ethyl methanesulfonate; GFP, green fluorescent protein; PDE, post-deirid; scm, seam cell marker; UTR, untranslated region.

Within this mapping interval, we found a 3 kb deletion in *vsc1* mutants located at the upstream region of the *lin-22* gene (Y54G2A.1) (Fig 2A). *lin-22* encodes a bHLH transcription factor that is related to Hes transcriptional repressors [37, 38]. Upon Sanger sequencing of that region, we found that the deletion extends to the 5′ end, deleting part of the last exon of the previous gene *vrp-1* (Y54G2A.3), and includes a 1.7 kb insertion aligning to sequences of the downstream gene *mca-3* (Y67D8C.10), collectively comprising the *icb38* mutation (Fig 2A and S1 Text). The following lines of evidence suggested that the *icb38* mutation is a new allele of *lin-22*; therefore, we refer to it as *lin-22(icb38)*. First, it has been previously shown that a hallmark phenotype in hermaphrodites upon disrupting *lin-22* function is an increase in the number of sensory post-deirid (PDE) neurons [37, 39, 40]. In WT, 1 PDE neuron is found per lateral side at the posterior body and is derived from the anterior V5 daughter following the first L2 division [13]. We found that *vsc1* mutants, similar to other *lin-22* mutants generated via Clustered Regularly Interspaced Short Palindromic Repeats/CRISPR-associated protein-9 (CRISPR/Cas9) genome editing or recovered from independent mutagenesis experiments also showed a significant increase in PDE number as monitored by an increase in *dat-1*∷*GFP* foci [41] (Fig 2B and S2A Fig). This included putative null alleles (*icb49*, *icb50* due to premature stop codons), another allele with an introduced stop codon in the third exon (*ot267*), as well as a mutation (*ot269*) of a single nucleotide located 4,940 bp upstream from the *lin-22* ATG (Fig 2A), in a region that is deleted in *vsc1* mutants, suggesting that this promoter region may play some regulatory role. Importantly, we found that all *lin-22* mutant alleles as well as RNA interference (RNAi) treatment targeting the *lin-22* gene and not the upstream gene *vrp-1*, led to an increase in seam cell number variability (Fig 2C and S2B–S2F Fig).

Importantly, seam cell number variability in *lin-22(icb38)* mutants was cell marker-independent as we observed a similar phenotype when using a *bro-1*∷*GFP* transgene to label the seam cells [17] (S2G Fig). We also used genome editing to engineer a putative null *lin-22* mutation in the CB4856 isolate and found a comparable increase in seam cell number variability and PDE number, suggesting that these phenotypes were independent of the N2 genetic background (S2H and S2I Fig). Seam cell number variability was sex-independent because it was also observed in males (S2J Fig), as well as lateral side-independent, as we found no correlation between seam cell counts obtained from one side of the animal to those for the other (S2K Fig).

### *lin-22* is expressed in seam cells anterior to V5

**Fig 3:**
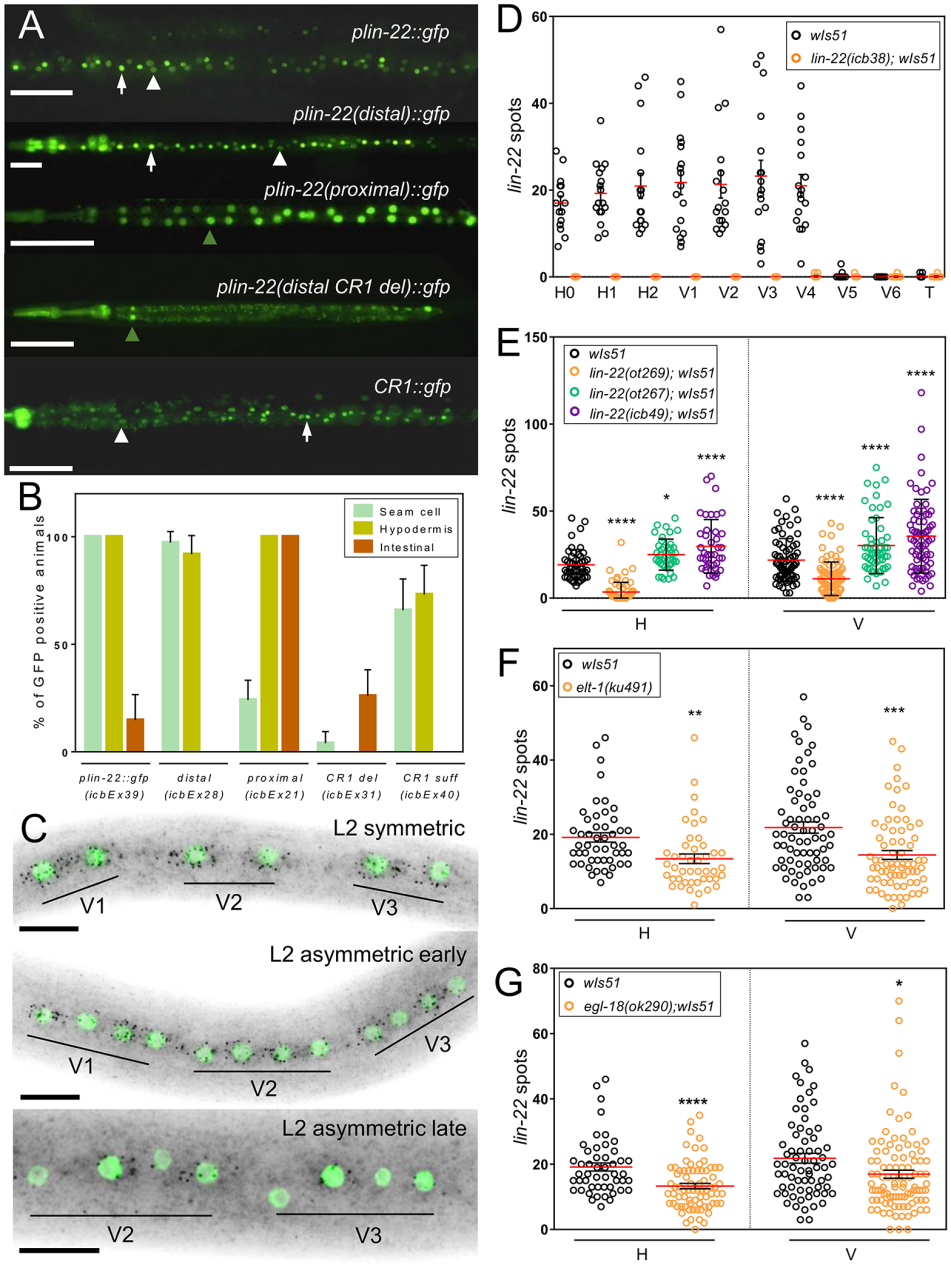
Quantification of *lin-22* expression in the seam. (A) Transgenic animals carrying transcriptional reporters consisting of various fragments of upstream of *lin-22* sequences fused to GFP. From top to bottom: full *lin-22* endogenous promoter, distal *lin-22* promoter region that is deleted in *lin-22(icb38)*, proximal *lin-22* promoter present in *lin-22(icb38)*, distal *lin-22* promoter with deleted CR1, and CR1 only driving expression of GFP. White arrows indicate expression in the seam cells; white arrowheads expression in the hypodermis and green arrowheads expression in intestinal cells. (B) Quantification of expression pattern for each transcriptional reporter (*n* ≥ 35 animals). (C) Representative smFISH images showing *lin-22* expression (black spots correspond to mRNAs and seam cells are labelled in green due to *scm*∷*GFP* expression) in wild-type V cells after the symmetric L2 division (top), the L2 asymmetric division (middle), and late after the L2 asymmetric division (bottom). (D) Quantification of *lin-22* spots per seam cell in wild-type and *lin-22(icb38)* animals at the late L1 stage (*n* ≥ 10 cells per genotype). (E) Quantification of *lin-22* spots in wild-type, *lin-22(ot267)*, *lin-22(ot269)*, and *lin-22(icb49)* mutants in pools of H cells and V cells at the late L1 stage (*n* ≥ 41). (F-G) Comparison of number of *lin-22* spots between wild-type and the *elt-1(ku491)* mutant (F) or the *egl-18(ok290)* mutant, (G) both at the late L1 stage in pools of H and V cells (*n* ≥ 49). Black stars show statistically significant changes in the mean with a *t* test or one-way ANOVA as follows: * *P* < 0.05, ** *P* < 0.01, *** *P* < 0.001, **** *P* < 0.0001. Reference samples for comparisons in E, F, G are the control samples depicted in black. Scale bars in A and C are 100 μm and 10 μm, respectively. Error bars show mean ± SEM (D, F, G) or mean ± SD (E). Numerical data used for Fig 3B, D, E, F, G can be found in S2 Data. CR1, conserved region 1; GFP, green fluorescent protein; L1, first larval stage; L2, second larval stage; smFISH, single molecule fluorescent in situ hybridization.

Because the *lin-22(icb38)* mutation mapped to the upstream noncoding region of *lin-22*, we went on to study aspects of *lin-22* promoter regulation and the spatiotemporal pattern of *lin-22* expression. To this end, we first constructed reporter fusions by placing either (1) the full *lin-22* promoter (approximately 5 kb), or (2) the distal to ATG *lin-22* promoter (approximately 3 kb) that is deleted in *lin-22(icb38)* mutants, or (3) the proximal *lin-22* promoter (approximately 2.2 kb) remaining in *lin-22(icb38)* mutants in front of green fluorescent protein (GFP). We found that the full promoter drove *lin-22* expression mostly in the seam and hypodermis (hyp7), but also to a much lesser extent in the intestine (Fig 3A and 3B). We also observed that the distal *lin-22* promoter drove GFP expression in seam cells and hypodermis, indicating that the deleted region in *lin-22(icb38)* mutants contained some putative seam cell enhancer activity (Fig 3A and 3B). The proximal *lin-22* promoter fusion showed rare GFP expression in the seam but more frequent expression in the intestine (Fig 3A and 3B). Interestingly, within the deleted *lin-22* promoter region in *lin-22(icb38)* mutants we identified 2 conserved regions: conserved region 1 (CR1) and conserved region 2 (CR2) between *C*. *elegans* and other related *Caenorhabditis* nematodes (S3A Fig). We showed that CR1, which contains at least 2 putative GATA binding sites (S3A Fig), was required to drive GFP expression in the seam (Fig 3A and 3B). CR1 was also partially sufficient to restore expression in seam cells as 65% of the animals showed some GFP expression in the seam (Fig 3A and 3B, *n* = 50), out of which 18% showed GFP expression in all seam cells that is fully reminiscent of the expression driven by the 3 kb distal fragment.

To study the endogenous *lin-22* expression pattern, we used single molecule fluorescent in situ hybridization (smFISH), which allows detection of single mRNAs, thus providing a quantitative account of gene expression [42]. In WT, we found that *lin-22* expression in the seam is restricted to H0–H2 and V1–V4 cells (Fig 3C and 3D and S3B Fig). After cell division, we found that daughter cells show initially comparable amounts of *lin-22* expression, both after the L2 symmetric and the subsequent asymmetric divisions (Fig 3C and S3C Fig). However, *lin-22* expression was maintained specifically in the posterior seam cell–fated daughter cell late after asymmetric divisions (Fig 3C and S3C Fig). In contrast to the WT pattern, *lin-22* expression was completely absent in *lin-22(icb38)* mutants in the seam at all developmental stages (Fig 3D and S3B Fig). Instead, we found stronger expression in the intestine (S3D Fig), which is consistent with the increased intestinal expression that we also observed with the proximal *lin-22* promoter∷GFP fusion. Remarkably, *lin-22* expression in the seam was decreased in the *lin-22(ot269)* mutant background in which a single base substitution disrupts a GATA site within the CR1 region at the distal promoter of *lin-22* (Fig 3E and S3A Fig). However, *lin-22* seam cell expression was increased in *lin-22(ot267)* and the putative null allele *lin-22(icb49)* (Fig 3E), indicating that LIN-22 may regulate its own expression via negative feedback.

To address whether epidermal GATA factors may be upstream regulators of *lin-22*, we quantified *lin-22* seam cell expression by smFISH in loss of function mutants of *elt-1* and *egl-18* [18,43] (Fig 3F and 3G) and *elt-1*/*egl-18* RNAi-treated animals (S3E Fig). In both cases, the number of *lin-22* spots detected was decreased. Taken together, our data suggest that the *lin-22(icb38)* mutation leads to loss of *lin-22* expression in the epidermis due to the deletion of a GATA site-containing enhancer that is found at the distal end of the *lin-22* promoter.

### Stochastic loss and gain of symmetric cell divisions underlie seam cell number variability in *lin-22* mutants

The developmental regulation of seam cell number relies on the right balance between epidermal cell proliferation and differentiation. These processes can be monitored by using the *scm*∷*GFP* transgene and lineage analysis, which relies on following GFP-positive cells and their patterns of division. When a *scm*∷*GFP* positive cell divides, the 2 daughter cells initially express GFP; however, GFP expression persists in the daughter cell that maintains the seam cell fate and disappears in the daughter cell that differentiates. Most V lineages contribute 2 seam cells to the terminal seam cell number, with the exception of V5 that contributes only 1, as it does not undergo a symmetric division at the early L2 stage (Fig 1A). Previous studies had shown that the increase in PDE number in *lin-22* mutant hermaphrodites stems from putative homeotic transformations of V1–V4 to that of V5 [37,40], leading to the prediction that *lin-22* mutants were likely to show a decrease in seam cell number as opposed to variability.

**Fig 4:**
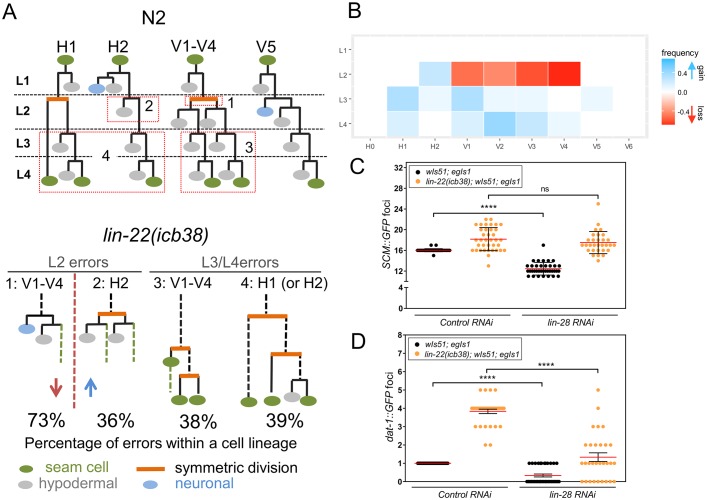
The developmental basis of variability in *lin-22* mutants. (A) The upper panel shows wild-type seam cell lineages, while the bottom panel indicates the most frequently occurring errors in *lin-22(icb38)* mutants (*n* = 14 independent complete lineages). The developmental errors are grouped for simplicity in 4 main classes and presented as a function of the developmental stage. The percentages refer to occurrence of these errors within the total number of relevant cell lineages. Note that the errors described are not independent, so they can occur within the same animal and even within the same lineage (see also S4 Fig). (B) Heat map showing the frequency of errors per cell lineage and developmental stage (*n* = 14 lineages). Blue depicts errors leading to gain of terminal cell number due to gain of symmetric division, and red depicts errors leading to loss of symmetric division. (C-D) Quantification of seam cell number (C) and number of PDE neurons (D) in wild-type (*n* ≥ 36) and *lin-22(icb38)* animals (*n* ≥ 30) treated with control or *lin-28* RNAi. Black stars show statistically significant changes in the mean with a *t* test (*P* < 0.0001). Error bars show mean ± SD (C) or mean ± SEM in (D). Numerical data used for Fig 4B, C, D can be found in S2 Data. L2, second larval stage; L3, third larval stage; L4, fourth larval stage; PDE, post-deirid; RNAi, RNA interference.

To decipher the developmental basis of the Vsc phenotype, we performed stage-specific phenotypic analysis using fixed animals carrying both the *scm*∷*GFP* and *dat-1*∷*GFP* markers. We observed that *lin-22(icb38)* eggs hatch with 10 seam cells as normal, indicating that the increase in variability happens postembryonically. Following the first L2 division, we found that at least 2, but up to 4, V1–V4 cells stochastically generate a neuroblast in all animals analysed (*n* ≥ 50), instead of dividing symmetrically to produce 2 seam cells as in WT (error 1 in Fig 4A). However, at the same time, we observed previously uncharacterised defects, namely animals showing occasional H2 symmetric divisions at the L2 stage (error 2) and V1-V4/H cell symmetric divisions at the third larval (L3) and fourth larval (L4) stage (errors 3, 4; Fig 4A), as opposed to the WT asymmetric divisions for these lineages at these developmental stages.

To verify these initial observations and establish complete seam cell lineages, we performed time-lapse microscopy by imaging *scm*∷*GFP* worms while growing from eggs to adults in custom-made microchambers and with 20 minute time resolution [44]. We found frequent V1–V4 asymmetric divisions at the early L2 stage, with the anterior daughter contributing ectopic PDE neurons instead of seam cells (in 32 out of 56 V1–V4 cell lineages analysed) (Fig 4A and 4B), although in some instances the anterior daughters gave rise to hybrid lineages contributing both neurons and seam cells (in 9 out of 56 V1–V4 lineages), as previously described [40]. Importantly, we validated the symmetric H2 divisions (in 5 out of 14 of H2 cell lineages) at the L2 stage. We also validated stochastic symmetric divisions for V1–V4 (in 21 out of 56 V1–V4 lineages) and H1/H2 cells (in 11 out of 28 H1/H2 cell lineages) at the L3 and L4 stage (Fig 4A and 4B and S4B Fig). Infrequently, we found single V1–V4 cell lineages repeating the L2 division pattern at the L3 stage (in 3 out of 56 V1–V4 lineages) or V5 and V6 symmetric divisions at the L3 and L4 stage (in 3 out of 28 V5–V6 lineages; S4 Fig). We conclude that the developmental basis of the cell number variability in *lin-22* mutants is the stochastic loss and gain of symmetric cell divisions. Notably, these developmental errors affect the terminal seam cell number in opposing directions and can co-occur within a single animal (S4A Fig) and cell lineage (Fig 4B).

The lineage analysis suggested that 2 sides of the seam cell number distribution could be partially separated in time, with ectopic neurogenesis occurring exclusively at the L2 stage and gain of seam cell fate happening mostly at the L3 and L4 stage. Therefore, we predicted that if animals were forced to skip the L2 stage, then seam cell number would decrease in WT due to loss of the L2 symmetric division, whereas gain of seam cell fate at the L3 and L4 stage would antagonise the loss of L2 symmetric divisions in *lin-22* mutants. To this end, we knocked down the expression of *lin-28*, a factor required for events specific to the L2 stage. In keeping with our prediction, we found that although seam cell numbers decreased in *lin-28* RNAi-treated WT animals, this decrease was suppressed in *lin-22(icb38)* mutants (Fig 4C). Instead, the number of ectopic PDE neurons, which is exclusively determined at the L2 stage, decreased in *lin-28* RNAi-treated both in *lin-22(icb38)* animals and the WT, as predicted (Fig 4D).

### Gene expression changes associated with loss and gain of daughter cell fate symmetry in *lin-22* mutants

**Fig 5:**
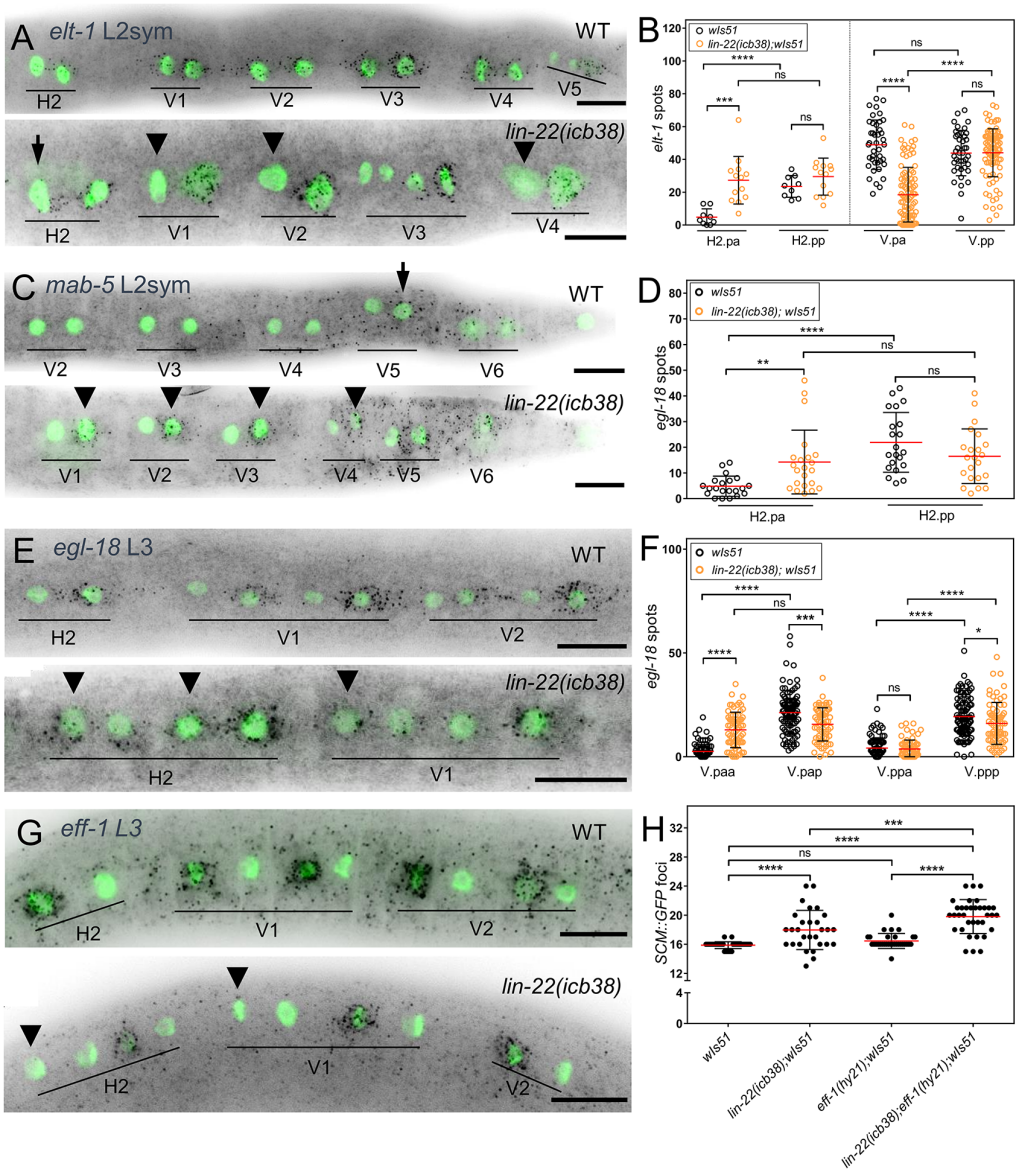
Gene expression changes associated with loss and gain of daughter cell fate symmetry in *lin-22* mutants. (A-B) Representative smFISH images and quantification of wild-type and *lin-22(icb38)* animals at the L2 symmetric division stage using an *elt-1* probe. (A) Comparable amounts of *elt-1* spots are detected in wild-type V cell daughters and more spots in the posterior H2 daughter. In *lin-22(icb38)* animals, more spots are detected in the posterior V daughters than the anterior (marked by arrowheads) and even numbers in the 2 H2.p daughters (arrow points to anterior H2.p daughter cell). (B) Quantification of *elt-1* expression in H2.p daughters (*n* > 9) and pools of V.p daughter cells (*n* > 41) of wild-type and *lin-22(icb38)* animals at the L2 symmetric division stage. (C) *mab-5* expression expands to posterior daughters of V1–V4 cells (arrowheads) in *lin-22(icb38)* animals, reminiscent of the expression in the posterior V5 cell in wild-type (arrow). (D-F) *egl-18* smFISH images and quantification of wild-type and *lin-22(icb38)* animals. (D) Quantification of *egl-18* smFISH spots in the H2.p cell daughters at the L2 stage (*n* ≥ 21). (E) Images depicting *egl-18* expression at the L3 stage. Note expression in anterior daughter cells in *lin-22(icb38)* animals (arrowheads). (F) Quantification of *egl-18* smFISH spots in V1-V4 cells (*n* ≥ 68) of wild-type and *lin-22(icb38)* animals at the L2 asymmetric division stage. (G) Representative *eff-1* smFISH images of wild-type and *lin-22(icb38)* animals at the L3 asymmetric division stage. Note absence of signal in the most anterior of the 4 daughter cells in *lin-22(icb38)* animals (arrowheads). H2 consists of 4 cells that have arisen due to symmetric division at the L2 stage. (H) Quantification of seam cell number in wild-type (*n* = 39), *lin-22(icb38)* (*n* = 29), *eff-1(hy21)* (*n* = 31), and *lin-22(icb38)*; *eff-1(hy21)* (*n* = 35) animals. Note that the *eff-1(hy21)* does not show a significant difference in seam cell numbers compared to wild-type, but the double mutant does in comparison to both parental strains. Black stars show statistically significant changes in the mean with a *t* test or one-way ANOVA /Dunnett’s test. Scale bars in A, C, E, and G are 10 μm; black spots correspond to mRNAs and green labels the seam cell nuclei. Error bars in B, D, F, H show mean ± SD. Numerical data used for Fig 5B, D, F, H can be found in S2 Data. GFP, green fluorescent protein; L2, second larval stage; L2sym, symmetric first division at the L2 stage; L3, third larval stage; SCM, seam cell marker; smFISH, single molecule fluorescent in situ hybridization.

We went on to explore gene expression changes that might be associated with the daughter cell fate symmetry defects as predicted based on lineage analysis. To this end, we studied the expression of key genes of the seam cell gene network by smFISH. We first detected the expression of the GATA transcription factor *elt-1* involved in epidermal cell specification. We particularly focused on H2 and V1–V4 cells, which are adjacent cells and yet display contrasting developmental errors at the early L2 stage—H2 often divides symmetrically in the mutant as opposed to asymmetrically, and V1–V4 divide asymmetrically in the mutant rather than symmetrically. Transcripts for *elt-1* were detected in WT animals in both the anterior and posterior V1–V4 daughters following the L2 symmetric division and mostly in the posterior daughters of H2 (Fig 5A and 5B). In stark contrast, we found that in *lin-22(icb38)* mutants the *elt-1* expression pattern in V1–V4 daughter cells became asymmetric with the posterior cells expressing more *elt-1* than the anterior (Fig 5A and 5B). At the same time, *elt-1* expression in H2 was found to convert to a symmetric pattern in *lin-22* mutants (Fig 5A and 5B), with anterior daughter cells in the mutant showing an increase in expression compared to the WT.

Another candidate we studied is the posterior Hox gene *mab-5*, previously shown to expand qualitatively to the anterior side in *lin-22* mutants [37, 45]. In WT, we detected expression at the posterior end of the animal and specific localisation to the posterior V5 daughter cell after the first L2 division (Fig 5C). In *lin-22(icb38)* mutants, *mab-5* signal expanded anteriorly but mRNA spots were detected in an asymmetric way in posterior V cell daughters and at comparable levels to expression in V5 (Fig 5C and S5A Fig). Therefore, with regard to both *elt-1* and *mab-5* expression, the V1–V4 gene expression pattern in *lin-22* mutants is reminiscent of that in V5. In both cases, the expression pattern change was very frequent (S5B and S5C Fig), yet variable among cells of a single animal, with some seam cells showing a change in pattern when adjacent cells did not (S5D Fig).

We further studied the expression of the GATA factor *egl-18*, which acts downstream of the Wnt pathway influencing seam cell fate [18]. In WT animals, *egl-18* is enriched in the posterior daughter cell following the L2 asymmetric division of H2 (Fig 5D), and also enriched in the posterior daughter cells following the subsequent V1–V4 asymmetric division (Fig 5E and 5F). In *lin-22(icb38)* mutants, we quantified an increase in *egl-18* expression in anterior H2.p daughter cells at the L2 division with 36% (8 out of 22 H2.pa cells) of *egl-18* expression values in *lin-22(icb38)* mutants outside the WT range (Fig 5D). Interestingly, this frequency of *egl-18* increase in expression approximately matches the frequency of cell fate symmetry observed in lineaging. At the subsequent asymmetric division, *egl-18* expression in the mutant also expanded with typically the most anterior of the 4 V cell daughters (V.paa) showing higher expression in the mutant as compared to the WT, with 16 out of 68 *egl-18* expression values in the mutant V.paa cells being outside the WT range (Fig 5E and 5F). Importantly, although this increase in V.paa *egl-18* expression was observed in the majority of the animals analysed (19 out of 20 animals), in most cases only some V cells were displaying this pattern (17 out of 19 animals), indicating again substantial cell-to-cell variability (S5D and S5E Fig).

Anterior daughter cell differentiation culminates in cell fusion to the adjacent hypodermal syncytium, a process that is mediated by the transmembrane fusogen protein EFF-1 [17,46]. In WT, we detected very tightly regulated *eff-1* induction in bursts localised to the anterior differentiating daughter cells (Fig 5G). Consistent with the stochastic loss of differentiation in *lin-22(icb38)* mutants and the increase in *egl-18* expression in the most anterior V cell daughters, we found frequent absence of *eff-1* expression in V.paa cells (19 out of 35 cells; Fig 5G) and enhanced fusion defects in *eff-1(hy21)*; *lin-22(icb38)* double mutants (Fig 5H). Taken together, these gene expression results highlight the loss and gain of molecular symmetry at different cell lineages and cell-to-cell variability in gene expression.

### *lin-22* mutants show variable increase in Wnt pathway activity in the seam

To understand further the mechanistic basis of seam cell number variability in *lin-22* mutants and the unexpected increase in seam cell number in particular, we performed gene expression analysis via RNA sequencing. Although we used whole animal tissue in these experiments, we hypothesised that we might be able to pin down some specific changes relevant to the epidermis due to the largely tissue-specific *lin-22* expression pattern. Validating the approach, cell fate transformations in *lin-22* mutants have been attributed to transcriptional de-repression of neuronal regulators [37], and we found that key neuronal development genes, such as the bHLH factors *lin-32* and *hlh-14*, were significantly upregulated in *lin-22* mutants (S1 Data and S6A Fig). Consistent with our smFISH results, we found a decrease in *lin-22* expression specifically in *lin-22* mutants that harbour mutations in upstream regulatory sequences (S6B Fig). We then focused on putative components of the seam cell gene network to identify gene expression changes that may have a direct influence on seam cell patterning (S6B Fig). Interestingly, we found changes in Wnt-related components in *lin-22* mutants, which led us to explore further a possible link between *lin-22* and Wnt signalling.

**Fig 6:**
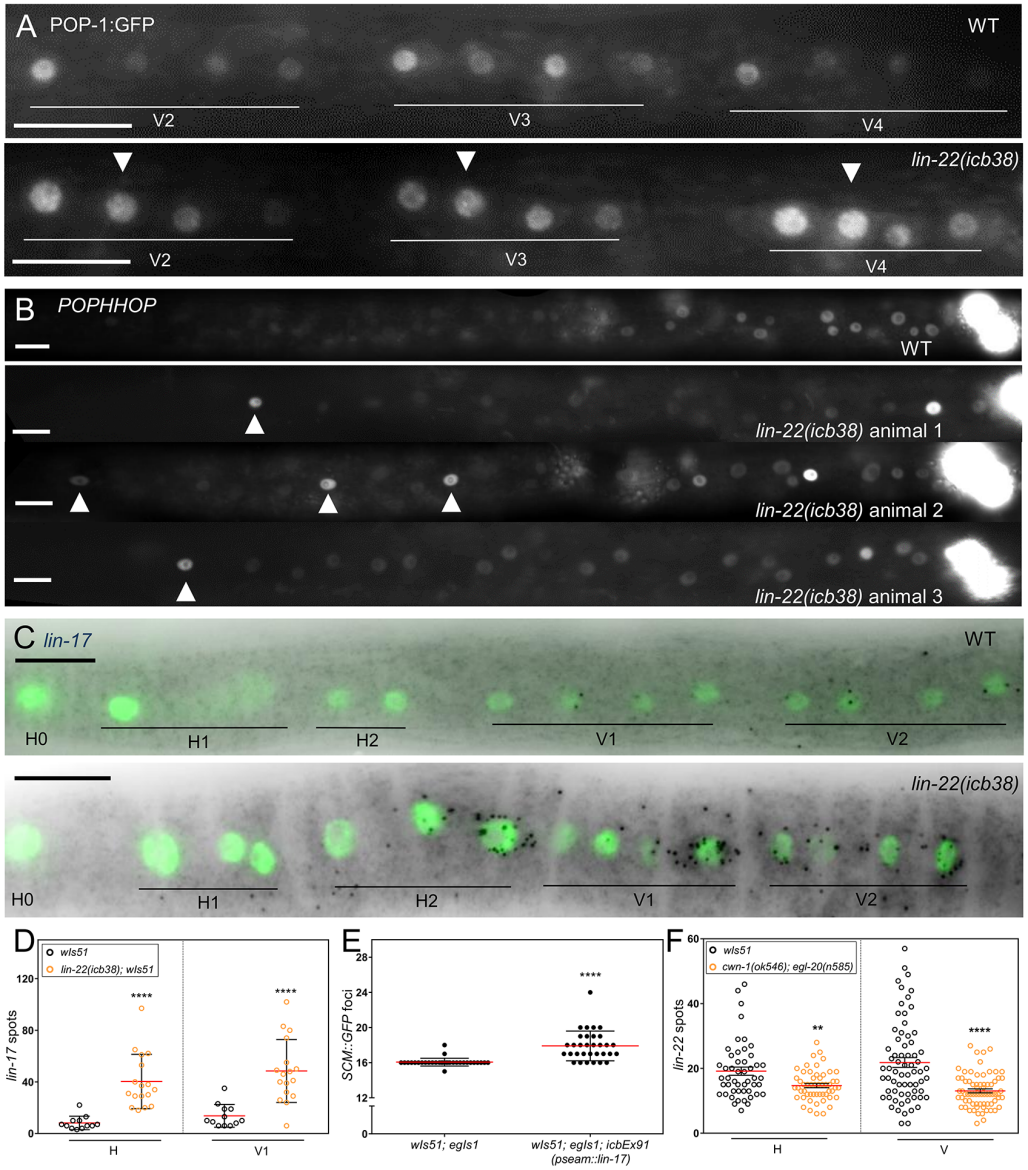
*lin-22* is both upstream and downstream of Wnt signalling. (A) Wild-type and *lin-22(icb38)* animals at the L2 asymmetric division stage carrying a POP-1:GFP translational reporter. Note aberrant polarity in *lin-22(icb38)* mutants (white arrowhead) and variable intensity (we found 50% of pairs showing comparable intensity to the wild-type posterior and 50% to the wild-type anterior cells). (B) Representative wild-type and selection of *lin-22(icb38)* animals at the L2 stage carrying the *POPHHOP* reporter. Note expression in more anterior seam cells in *lin-22(icb38)* mutants (white arrowheads). (C) Representative *lin-17* smFISH images of the anterior body of wild-type and *lin-22(icb38)* animals at the L3 division stage. (D) Quantification of *lin-17* smFISH spots (in black) in V1 and H cells (labelled with GFP) of wild-type (*n* = 12) and *lin-22(icb38)* (*n* = 17) animals at the L3 asymmetric division stage. (E) Quantification of seam cell numbers in wild-type animals expressing *lin-17* under a seam cell-specific promoter. (F) Quantification of *lin-22* smFISH spots in wild-type and the Wnt ligand double mutant, *cwn-1(ok546);egl-20(n585)* at the late L1 stage in pools of H (*n* > 49) and V cells (*n* > 68). Black stars show statistically significant changes in the mean with a *t* test (reference sample in D, F is *wIs51* depicted in black). Scale bars are 10 μm. Error bars show mean ± SD (D, E) or mean ± SEM (F). Numerical data used for Fig 6D, E, F can be found in S2 Data. GFP, green fluorescent protein; L2, second larval stage; L3, third larval stage; POPHHOP, POP-1 and HMG-helper optimal promoter; SCM, seam cell marker; smFISH, single molecule fluorescent in situ hybridization.

To address this possibility, we first compared POP-1 localisation between *lin-22* mutants and the WT. POP-1, which is the T-cell factor/lymphoid enhancer factor (TCF/LEF) homolog in *C*. *elegans* [47], controls multiple asymmetric divisions during embryo and larval development [48,49]. POP-1 has been shown to asymmetrically localise in seam cell divisions with anterior nuclei containing more POP-1 than SYS-1, leading to unbound POP-1 repressing Wnt-regulated genes, while posterior signalled cells exhibit less localisation to the nucleus, so that all POP-1 is bound to SYS-1 complex, which activates target genes [19,50]. We imaged POP-1:GFP localisation at the 4 V cell stage in L2 asymmetric division. For each WT cell pair, we found pronounced nuclear localisation at the anterior daughter nucleus (a) (Fig 6A), with only 6.6% of pairs showing aberrant localisation such as equal GFP intensity between the 2 daughter cells (5.3%) or enrichment at the posterior nucleus (p) (a < p in 1.3%; *n* = 76). However, we found that 30.1% of daughter V pairs in *lin-22(icb38)* mutants showed aberrant POP-1:GFP polarity (a = p in 21.9% and a < p in 8.2%; *n* = 146 and Fisher’s test *P* value < 0.0001; Fig 6A).

To monitor Wnt pathway activity directly, we introduced the POP-1 and HMG-helper optimal promoter (POPHHOP) marker in *lin-22(icb38)* mutants [51]. This marker reports POP-1 binding to a synthetic enhancer and is strongly expressed around the tail and mildly in the most posterior seam cells [51]. Interestingly, *lin-22(icb38)* mutants displayed an expansion of Wnt pathway activity with anterior to somatic gonad seam cells, including H cells, frequently expressing the marker (10 out of 21 animals in the mutant as opposed to 2 out of 30 in the WT, Fisher’s test, *P* value < 0.01) (Fig 6B). Interestingly, the expansion in the POPHHOP expression domain was sporadic and not observed in a graded manner from the highly expressing cells in the tail to the head of the animal. This indicates cell-to-cell variability in Wnt pathway activation along the body (Fig 6B).

To address whether this variability in Wnt pathway activity may have any phenotypic consequences for the seam, we sought to establish a correlation between POPHHOP marker activation and seam cell fate. Due to technical limitations, we were unable to follow cells expressing the marker using time-lapse microscopy. However, we focused on the activation of the marker in H cells and a partially penetrant yet distinctive phenotype in *lin-22* mutants, which is the presence of supernumerary seam cells in the head region in around 40% of the animals, likely due to symmetric divisions increasing the seam cell pool in that area (S5F Fig). We asked whether animals that show ectopic POPHHOP expression in H cells at the L2 stage are more likely to develop head seam cell clusters. We found that *lin-22(icb38)* animals selected for POPHHOP marker activation in the head are more likely to show this phenotype compared to animals not expressing the marker or animals selected at random (S5G Fig), thus variable Wnt pathway activation in *lin-22* seam cells may be directly linked to changes in cell fate.

To explore further the changes in Wnt pathway activity, we compared the expression of Wnt ligands and receptors between *lin-22(icb38)* and WT animals. We found evidence that the Wnt receptor *lin-17*, which is normally expressed only at the posterior end of the animal at the L3 stage, is ectopically induced in more anterior H and V seam cells in *lin-22(icb38)* mutants (Fig 6C and 6D and S5H Fig). We hypothesised that an expansion in *lin-17* expression may lead to more cells receiving Wnt ligands, thus acquiring the seam cell fate. To test this hypothesis, we produced transgenic animals expressing *lin-17* under a seam cell promoter and showed that this transgene is sufficient to cause an increase in seam cell number and variance, although the latter is likely to be purely technical due to the unstable nature of the transgene arrays (Fig 6E). Consistent with the decrease of *lin-22* expression in *egl-18* loss of function mutants, we obtained further evidence that *lin-22* also acts downstream of the Wnt pathway, as *lin-22* expression was mildly decreased in the double Wnt ligand mutant background *cwn-1(ok546); egl-20(n585)*, which is aphenotypic for seam cell number (Fig 6F) [52]. Taken together, our data provide support for a novel crosstalk between *lin-22* and Wnt signalling.

### *lin-22(icb38)* mutants show defects mainly at the epidermis

**Fig 7:**
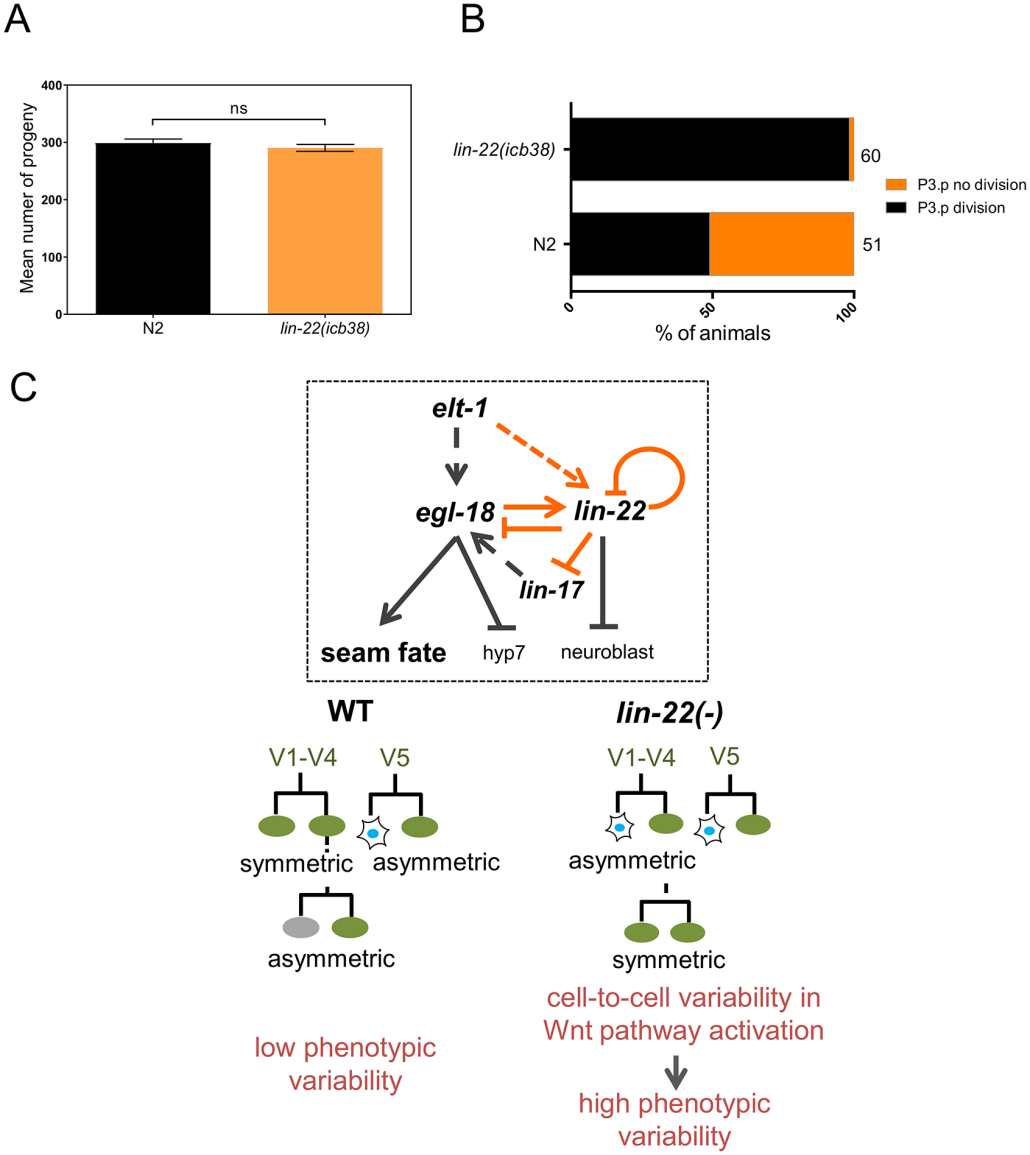
Context-dependent gain and loss of variability in *lin-22(icb38)* mutants. (A) Quantification of brood size in wild-type (*n* = 13) and *lin-22(icb38)* mutants (*n* = 15). Black bars show mean ± SEM. (B) Quantification of P3.p division frequency in wild-type and *lin-22(icb38)* animals. Note that almost all mutant animals show division of P3.p. (C) Model showing *lin-22* interactions discovered in this study (orange), while previously known interactions are shown with dashed grey lines. These new interactions may not be direct. Seam cell number variability is increased in *lin-22* mutants due to loss and gain of symmetric divisions. Stochastic loss of symmetric divisions at the L2 stage generates more neuroblasts at the expense of seam cells. Stochastic gain of symmetric divisions towards the seam cell fate mostly at the L3/L4 stage generates more seam cells. Cell-to-cell variability in Wnt pathway activation correlates with phenotypic variability. Numerical data used for Fig 7A, B can be found in S2 Data. L2, second larval stage; L3, third larval stage; L4, fourth larval stage.

It is possible that regulators identified in our seam cell screen might also increase variation for a number of independent phenotypes, which might be indicative of loss of animal fitness [23,33]. To test whether *lin-22* mutants show any fitness defect, we quantified brood size in *lin-22(icb38)* animals and found no statistically significant difference to the WT N2 (Fig 7A). To assess tissue specificity of the phenotypic variability, we looked into other developmental decisions involving tight control of cell numbers in *C*. *elegans*. One case of natural variability in cell numbers in the WT concerns P3.p, which is the most anterior vulval cell that divides once before fusing with the epidermis in around 50% of the animals. We compared P3.p division frequency between the WT and *lin-22(icb38)* and found that P3.p division occurs in nearly 100% of *lin-22(icb38)* mutants (Fig 7B). We then quantified by differential interference contrast (DIC) microscopy the number of Pn.p cells induced to acquire vulval fates and found a very mild increase in *lin-22(icb38)*, mainly due to low penetrant P3.p and P4.p induction (S7A Fig). We also quantified the number of uterine π cells using *lin-11*∷*GFP* as a marker [53] and found no difference between *lin-22(icb38)* mutants and the WT (S7B Fig). Last, we quantified the number of intestinal cells using an *elt-2*∷*GFP* marker [54] and found a marginal decrease in the number of nuclei in *lin-22(icb38)* mutants compared to the WT (S7C Fig). We conclude that *lin-22(icb38)* mutants show an increase in phenotypic variability predominantly in the seam.

## Discussion

### *lin-22* and single genes modulating variance

In this study, we performed a targeted genetic screen in *C*. *elegans* to identify factors shaping phenotypic variance. In particular, we screened for mutants showing an increase in epidermal seam cell number variability without a change in the mean. We identified a deletion in the distal promoter of the transcription factor *lin-22* as the molecular cause of seam cell number variability. We showed that this deletion removes a seam cell enhancer and, thus, attenuates *lin-22* expression to the extent that we could no longer detect any transcript in the seam. Consistent with this finding, the recovered mutant phenocopies other *lin-22* null alleles with respect to seam cell number variability.

Identifying genes that modulate (enhance or suppress) phenotypic variance in a given developmental system is a fundamental problem in biology that has implications for disease and drug discovery [55]. A key question is whether variance modulators are integrated within developmental gene networks or they are superimposed as core cell homeostasis factors influencing variance. There are examples in the literature supporting the latter possibility with the most prominent being the molecular chaperone *HSP90*, which is thought to suppress variation for a variety of different phenotypes [9,56]. More high-throughput screens in yeast for genes buffering morphological variation have also identified chromatin factors, cell cycle proteins, components of stress response, and essential genes as key components influencing variability [6,23]. In a recent example in plants, a mutation in a broadly expressed mitochondrial protease was found to increase variability in sepal size and shape [57]. In this case, organ shape uniformity was shown to arise from spatiotemporal averaging of already variable cellular growth in WT.

Our seam cell number variability screen identified a transcription factor, which we placed within the seam cell gene network (Fig 7C). Consistent with our definition of genes modulating variability (Box 1), *lin-22* null mutants show pronounced phenotypic variability in the seam with 2-sided phenotypic errors and no change in the mean. This phenotypic variability is tissue specific, with no evidence for systemic defects; therefore, it is unlikely to be driven by animal sickness. Interestingly, we also demonstrate that the recovered *lin-22* mutation and other *vsc* mutants have more pronounced effects on variability than impairment of *Hsp90*. Our ability to isolate *vsc* mutants is consistent with theoretical work that suggests modulators of variance may be widespread in developmental systems [58].

Related Hes bHLH proteins act in mammals as transcriptional repressors and play a role in the maintenance of stem cells and progenitors in neural and digestive organ development, influencing binary cell fate decisions [38]. They are also relevant to disease as they are thought to maintain the stemness of cancer stem cells [59]. Stochastic variation in the expression of *Hes1* in mouse embryonic stem cells influences neuronal versus mesodermal differentiation and contributes to heterogeneous cell responses such as the timing of commitment of pluripotent stem cells to differentiate [60,61]. In comparison to canonical HES factors, LIN-22 does not physically interact with the Groucho homologue UNC-37 as it is lacking a Groucho interacting domain [62]. Therefore, it may rely on passive repression mechanisms by competing for binding sites with other bHLH activators.

### The developmental basis of seam cell number variability in *lin-22* mutants

By using a combination of molecular genetics and time-lapse imaging, we studied the underlying developmental basis of phenotypic variability. We confirmed previous observations that *lin-22* mutants [37, 40], like mutants in related transcription factors in other systems [63], show extensive ectopic neurogenesis, which in our model correlates with anterior to posterior lineage transformations. More specifically, V1–V4 cells normally undergoing symmetric cell divisions at the early L2 stage in the WT divide asymmetrically in *lin-22* mutants similar to V5, with the anterior daughter cell generating a neuroblast. At the same stage, H2 cells often divide symmetrically in *lin-22* mutants in a manner that resembles V1-V4 lineages of WT, giving rise to daughter cells that do not fuse to the hypodermis and retain seam cell potential. These cell lineage transformations are stage specific, because at subsequent developmental stages both H and V cells can divide symmetrically in *lin-22* mutants in a pattern that is not seen in WT lineages at this stage. Interestingly, both types of developmental errors occur within a single *lin-22* mutant animal and even within the very same cell lineage but at different stages, possibly relying on the availability of other factors that contextualise the *lin-22* role. However, these 2 trends show variable expressivity, therefore, which cells generate neuroblasts or show aberrant symmetric divisions varies stochastically in the population. This developmental tug-of-war between loss and gain of symmetric divisions in *lin-22* mutants results in lineages losing and/or gaining seam cells, thereby pushing the terminal seam cell number to either side of the population average of 16 cells.

In particular, we explored the mechanistic basis of the symmetrisation of divisions in *lin-22* mutants, which was a previously unknown phenotype. We showed that *lin-22* mutants feature a hyper activation of Wnt pathway in the seam, as is evident from the increased expression of the downstream target *egl-18* in anterior daughter cells, which may act to prevent seam cell daughter differentiation to hyp7 [19]. We also found an increase in the expression of the Wnt receptor *lin-17*, previously known to modulate asymmetric cell divisions [19,52,64], and a Wnt pathway activity reporter. In the Q neuroblast, the *lin-17* receptor itself has been shown to be a transcriptional target of the Wnt pathway [65]; therefore, it is possible that *lin-17* upregulation in *lin-22(icb38)* mutants is either a cause or consequence of Wnt pathway activation. The Wnt pathway acts throughout the nervous system in *C*. *elegans* [66] so it is likely that the activation of Wnt also facilitates the ectopic neurogenesis observed in *lin-22* mutants.

Interestingly, the spatial activation of the Wnt pathway was found to be variable in *lin-22* mutants, with some seam cells showing strong expression of the Wnt pathway reporter when adjacent seam cells did not. We propose that this stochasticity in Wnt pathway activation in *lin-22* mutants may drive phenotypic variability. We were able to establish a correlation between Wnt pathway activation in head seam cells at the L2 stage and the subsequent development of head seam cell clusters in the *lin-22* mutant background. In the future, with the advent of improved markers to visualise Wnt singalling, it will be very interesting to follow cells while they develop and establish direct correlations between Wnt pathway levels and cell fate. It will also be exciting to explore the underlying mechanisms of cell-to-cell variability in Wnt pathway activation in the seam via identifying missing Wnt pathway regulators and dissecting tissue-specific pathway feedback.

### Gene expression changes in the seam and link to variability

Previous studies in seam cell development have largely relied on reporter constructs that provide qualitative information. In our study, we have used smFISH for the first time in the seam to demonstrate that *lin-22* is specifically expressed in H0–H2 and V1–V4 cells, with a clear boundary between V4 and V5 that is consistent with its anterior developmental role. However, we observed that the full *lin-22* promoter fusion drives expression in all seam cells including the posterior V and T cells. This may be due to posttranscriptional regulation absent in the promoter construct; for example, some miRNA regulation as previously described for related bHLH factors in mice [67] or simply due to the multicopy nature of transgenesis in *C*. *elegans*. This highlights the importance of studying the endogenous mRNA expression in comparison to transcriptional reporter transgenes. The *lin-22* expression pattern also suggests that other developmental factors should act locally in V6 and T cells to inhibit neurogenesis [40].

Furthermore, we found *lin-22* expression to be dynamic during seam cell development, showing initially equal expression in both daughter cells post asymmetric division. This is different to the expression of *egl-18* and *elt-1*, both found to be enriched at the posterior seam-fated cell postdivision [18]. Interestingly, the expression of certain *Hes* genes oscillates in many cell types and *Hes* genes regulate the timing of critical biological events such as somite segmentation or the timing of neuronal differentiation [38,68]. A key aspect for *Hes1* expression oscillation is its rapid degradation and negative feedback from HES1 protein [69,70], with the latter being a feature in *lin-22* regulation as well. Such negative autoregulation is thought to provide stability to gene networks [71] and may be important to constrain *lin-22* expression variability. To better understand gene expression dynamics and expression pattern periodicity in the seam, it would be intriguing in the future to explore a possible connection with the heterochronic gene pathway [20,72].

The expression pattern changes we describe in *lin-22* mutants support lineage-specific loss and gain of daughter cell fate symmetry at the molecular level occurring within single animals. A striking example is the H2.p daughter cells in *lin-22* mutants, which shift from an asymmetric towards a symmetric *elt-1* and *egl-18* expression pattern, while the adjacent V1–V4 cells show the opposite trend. Interestingly, the gene expression changes we describe in *lin-22* null mutants are also subject to stochasticity, displaying cell-to-cell variability within a single animal. A key question is to identify how gene expression variability might relate to phenotypic variability [73, 74]. By comparing the frequency of gene expression defects and the frequency of aberrant cell linages, it appears once more that stochasticity in downstream Wnt signaling is likely to contribute to cell fate changes. For example, the increase in *egl-18* expression in H2.pa daughters is observed at a comparable frequency to the adoption of seam cell fate for these anterior daughters. On the other hand, expression in H2.pa of the upstream gene *elt-1* is increased more frequently than the observed cell fate symmetrisation, which may reflect a higher threshold for downstream Wnt pathway activation. Therefore, the smFISH results together with the Wnt reporter analysis suggest that stochasticity in Wnt pathway activation among seam cells in *lin-22* mutant animals may be an important component of the observed phenotypic variability (Fig 7C).

### Studying developmental variability in a systemic way

Over the last years, *C*. *elegans* has been used as a system to quantify the limits of developmental robustness to environmental variation and other perturbations [75–77]. There are several reasons why we decided to pursue genetic screens in the seam. First, different tissues might show different levels of sensitivity to perturbations. Seam cell number is sensitive to stochastic noise; therefore, we reasoned that the increased flexibility of seam-cell patterning would facilitate our efforts to identify mutations increasing trait variance [78]. Another key reason is that phenotyping in the seam is based on fluorescent markers, thus, it is amenable to high-throughput approaches including fluorescence-based animal sorting.

It is possible that regulators buffering seam cell number variability act cell autonomously within the seam or influence seam cell behaviour from a distance. Therefore, it will be interesting to explore systemic defects in mutants we recover from our screen. Despite some mild defects in the intestine, *lin-22* mutants have a normal brood size and in general there is no other tissue in which we could detect an increase in phenotypic variability as strikingly as in the seam. Consistent with this, seam cell defects in *lin-22* mutants were shown to be lateral side-autonomous. Therefore, it is unlikely that variability in the mutant is determined at the organismal level or comes as a side effect of loss of animal fitness. Remarkably, we found that although seam cell number is more variable in *lin-22* mutants, P3.p division frequency becomes less stochastic with almost all animals showing dividing P3.p cells that do not fuse to hypodermis at the L2 stage. Interestingly, Wnt pathway activation has been previously shown to result in almost 100% P3.p division frequency [79]. Therefore, consistent hyper activation of the Wnt pathway in P3.p in *lin-22* mutants or higher sensitivity, or some independent posterisation towards a P4.p fate, which always divides in WT, may explain the effect on P3.p division frequency. Nevertheless, this highlights that a single mutation may lead to either more variable or more deterministic events in different cell lineages (Fig 7C).

It remains a great challenge to dissect all mechanisms of phenotypic variability in multicellular systems and develop a developmental framework towards interpreting such phenotypes. Recent evidence suggests that multiple developmental decisions including stem cell patterning are governed by chance to some degree and buffering mechanisms are needed to operate at the cell or tissue level [80]. We anticipate that by cloning a broad spectrum of mutants derived from our screen and dissecting the underlying mechanisms we will increase our understanding on the genes modulating variance and their relationship to core developmental networks.

## Materials and methods

### Nematode culture and genetics

The strains used in this study were cultured and handled according to standard protocols [81]. The JR667 strain containing the *scm*∷*GFP* transgene (*wIs51*) is used as a reference on standard NGM plates with OP50 as a food source. A complete list of strains used in this study is presented in S1 Table.

### Genetic screen and mapping

EMS mutagenesis was performed according to standard procedures [81]. We screened 30,000 haploid genomes to recover seam cell number mutants. Briefly, nematodes were mutagenised in 4 ml total volume of M9 supplemented with 50 mM EMS (Sigma Aldrich, St. Louis, MO) with occasional rotation, then washed 10 times and plated for 1 hour to recover. F2 animals with extreme seam cell counts were selected either using a worm sorter at a speed of 10 animals per second (Union Biometrica, Holliston, MA) or manually under a stereomicroscope (Axio Zoom; Zeiss, Oberkochen, Germany) using CO_2_ and the following set up to immobilise animals: a 150 ml conical flask contained in a Styrofoam box was filled with 50–100 ml of absolute ethanol, and a 5 mm diameter rubber tube was fitted on a petri dish lid and the other end was connected to the flask. Dry ice was added until the temperature of the ethanol equilibrated and a constant flow of gas CO_2_ was achieved. Whole genome sequencing was performed using various Illumina platforms at 20- to 30-fold genome coverage and mapping was performed using the Cloudmap pipeline on Galaxy [82]. *lin-22(icb38)* was backcrossed 4 times before phenotypic characterization. The *ot269* mutation is a C to T change at −4,940 from *lin-22* ATG (TTTTAT**C**TTGATTTACGTGT). The *icb49* and *icb50* alleles are deletions of a single (ATTGAATCCG-TGGTGGAATCTC) or 5 nucleotides (ATTGAAT-----GGTGGAATCTC) within the first exon of *lin-22*. The *ot267* is a G to A change within the third exon (TCCAAATG**G**GAAAAAGCT). The *icb49*, *icb50* alleles lead to early stop codons, so we refer to them as putative null. The *icb49 lin-22* allele was also recovered in CB4856 through independent injections of the same sgRNA targeting *lin-22* in N2.

### Microscopy and phenotypic characterisation

For light and fluorescence microscopy, animals were mounted on 3% agar pads in M9 containing 100 μM sodium azide (NaN3), covered with a coverslip and viewed under an epifluorescence Ti-eclipse (Nikon, Minato, Tokyo, Japan) microscope. Seam cell and PDE neuron numbers were scored at the early adult stage using 1 lateral side per animal. Lineaging analysis was performed by synchronising animals containing both the *scm*∷*GFP* and *dat-1*∷*GFP* markers by egg laying over a period of 1 hour and observing them at different time points using epifluorescence microscopy. Time-lapse microscopy was performed as previously described [44]. For the POPHHOP selection experiment (S5F Fig), *lin-22(icb38)* mutants carrying the POPHHOP reporter and a red seam cell marker were synchronised by bleaching. Individual L2 animals were mounted on 5% agarose pads, anesthetized by using 10 mM muscimol and classified based on presence or absence of nuclear GFP signal in H cells, while keeping track of the lateral side by using the rectum as a reference. Animals were then grown individually and scored at the early adult stage for seam cell number and presence of H seam cell clusters.

### Single molecule fluorescent in situ hybridization

Synchronised nematode populations were produced by bleaching. Animals were fixed at the appropriate stage as directly monitored by microscopy and smFISH was performed as previously described [76] using a pool of 25–48 oligos fluorescently labelled with Quasar 670 (Biosearch Technologies, Novato, CA). Imaging was performed using a motorized epifluoresence Ti-eclipse microscope (Nikon) and a DU-934 CCD-17291 camera (Andor Technology, Belfast, United Kingdom) acquiring 0.8 um step z-stacks. Image analysis and spot quantification were performed on raw data using a MATLAB (MathWorks, Natick, MA) routine as previously described [76]. For the images presented in the results section of this study, the probe signal channel was inverted for clarity (black spots correspond to mRNAs) and merged to the seam cell or DAPI fluorescence channel using ImageJ (NIH, Rockville, MD). A complete list of smFISH oligo probes is presented in S2 Table.

### RNA-seq analysis

Larvae were synchronized by bleaching and grown to L3 stage (31 hr posthatching) before total RNA was extracted using TRIzol (Invitrogen, Carlsbad, CA) reagent. RNA quality was determined using the Agilent RNA ScreenType System on a 2100 Bioanalyzer (Agilent, Santa Clara, CA). The library preparation was done using the TruSeq stranded mRNA library preparation kit (Illumina, San Diego, CA). The sequencing data were processed and aligned to *C*. *elegans* reference genome using Bowtie2 [83]. The bam files were used to generate counts using bedtools [84]. The counts were then normalised using DESeq package in R [85]. Differences in gene expression were then calculated using the negative binomial test in the DESeq package (FDR = 0.1). RNA-seq data are deposited in the NCBI GEO under accession GSE101645.

### Genome editing

To edit the *lin-22* coding region, the co-CRISPR strategy was used [86]. An sgRNA targeting the following sequence (ACTGAAATTGAATCCGATGG) in the first exon of *lin-22* was cloned into *pU6*∷*unc-119_sgRNA* vector by replacing the *unc-119* sgRNA as previously described [87]. The injection mix contained *peft3*∷*cas9* at 50 ng/μl, *pU6*∷*dpy-10_sgRNA* at 25 ng/μl, *pU6*∷*lin-22_sgRNA* at 25 ng/μl, repair oligo template for *dpy-10* at 10 pmol/μl, and *myo2*∷*dsRed* at 5 ng/μl. F1 animals showing morphological phenotypes indicative of modifications at the *dpy-10* locus were examined for presence of multiple PDE neurons. PCR was performed on the F2 animals by using primers lin22-23F/lin22-22R, and the amplified fragment was sequenced to find the nature of the induced mutation.

### Cloning

To construct the *lin-22* promoter GFP reporters, the following cloning strategy was used. For the full promoter (*plin-22*∷*gfp*), a 5199 bp sequence upstream of the *lin-22* ATG was amplified by using the oligos lin22-1F and lin22-2Rfusion from fosmid WRM0627dG07. For the proximal promoter (*plin-22(proximal)*∷*gfp*), a 2,180 bp sequence upstream of the *lin-22* ATG was amplified by using the oligos lin22-3F and lin22-2Rfusion from the same fosmid. Both amplicons were fused by PCR to *GFP*∷*H2B*∷*unc-54* 3′ UTR amplified previously from a suitable plasmid using oligos GFP-F and unc54-R. Both constructs were injected into N2 animals at 10 ng/ul with *myo-2*∷*dsRed* as co-injection marker. For the distal *lin-22* promoter (*plin-22(distal)*∷*gfp*), the distal 3040 bp *lin-22* promoter, deleted in *lin-22(icb38)*, was amplified from the same *lin-22* containing fosmid using the primers lin22-17F and lin22-18R carrying restriction sites for StuI and NheI respectively. The amplicon was cloned in the L3135 vector (Addgene, Cambridge, MA) as a StuI/NheI fragment creating pDK1.

To create the CR1 deletion (*plin-22(distal CR1 del)*∷*gfp*) reporter, pDK1 was used as template to amplify 2 distinct fragments of the distal promoter, excluding the CR1 using primer pairs lin22-17F/ lin-22 _fus_CR1delR and lin-22_fus_CR1delF/lin22-18R. The 2 amplicons were fused by PCR and inserted into L3135. To create CR1 sufficient (*CR1*∷*gfp*) GFP reporter, CR1 was amplified from pDK1 by using primers lin-22_CR1REF and lin-22_CR1RER carrying compatible restriction sites and the amplicon was cloned in L3135. All 3 reporters were injected into N2 animals at 10 ng/μl with *myo-2*∷*dsRed* as co-injection marker.

To construct a vector to allow seam cell transgene expression, we used the last intron of *arf-3* (*arf-3i*) that is contained within the original pMF1 plasmid and is sufficient for seam cell expression. *arf-3i* and unc-54 were amplified from previously made plasmids using primer pairs arf-3-EcorI/pes-10-R-Fusion and unc-54-F-Fusion/ unc-54-HIII respectively. The 2 amplicons carried a fusion overlap, including a 23 bp sequence tag containing SwaI and PmeI restriction sites, and were fused by PCR. The resulting amplicon was cloned into pUC57 as an EcoRI/HindIII fragment producing pIR5. To express *lin-17* in the seam, *lin-17* was amplified from N2 cDNA using primers lin-17a3-p10 F and lin-17u54R, which carried compatible sequences and allowed insertion of the amplicon in a SwaI digested pIR5 via Gibson assembly. The resulting plasmid pDK5[*pseam*∷*lin-17*∷*unc-54 3’ UTR*] was injected at 30 ng/μl with *myo-2*∷*dsRed* as co-injection marker.

All constructs used in this study were verified by sequencing and at least 2 independent transgenic lines were obtained and compared. A list of all oligos used in this study is presented in S3 Table.

### RNAi

Animals were fed with dsRNA expressing bacteria as a food source. Bacteria were grown overnight and then seeded directly onto NGM plates containing 1 μM IPTG, 25 μg/ml ampicillin and 6.25 μg/ml tetracycline. To construct a *lin-22* RNAi feeding vector, *lin-22* was amplified using the TOPO cloning compatible lin22-15F primer and lin22-14R from the fosmid WRM0627dG07 (Source Bioscience, Nottingham, United Kingdom). The amplicon was inserted by TOPO cloning in pDONR/D-TOPO vector, creating the entry vector pENTR *lin-22*. The pENTR *lin-22* plasmid was used to insert *lin-22* in a gateway compatible L4440 vector via an LR reaction. To construct a *vrp-1* RNAi feeding vector, *vrp-1* was amplified from N2 genomic DNA using primers Y54G2A.3a F1 and Y54G2A.3a R1, first cloned in a pDNR/D-TOPO vector (Invitrogen, Carlsbad, CA) and then inserted into a gateway compatible L4440 vector. Both feeding vectors were transformed into *Escherichia coli* HT115 to be used for nematode feeding. The *elt-1* RNAi feeding vector is pAW565 as described in [17]. All other clones used in this study are commercially available from Source Bioscience.

## Supporting information

S1 Text Sequence file showing the *lin-22(icb38)* deletion and the position of CR1 and CR2 elements. CR1, conserved region 1; CR2, conserved region 2.
(DOCX)

S1 Data List of genes differentially expressed in *lin-22* mutants. (XLSX)

S2 Data Numerical data that are used in figures. (XLSX)

S1 Table List of strains used in this study. (XLSX)

S2 Table List of smFISH probes used in this study. (XLSX)

S3 Table List of oligos used in this study. (XLSX)

S1 Fig Mapping variable mutations (related to Fig 1). (A) Down-regulation of *Hsp90/daf-21* leads to marginal seam cell number variability in the seam (*n* ≥ 40). Red star depicts change in variance with a Levene’s median test (* *P* < 0.05). Error bar shows mean ± SD. (B) Graph showing the selected recombinant lines with CB4856 based on quantitative phenotyping of seam cell number standard deviation (SD), percentage of animals with extreme seam cell counts, and percentage of animals with 16 seam cells. Each circle represents 1 line. The parental *vsc1* mutant strain is depicted in red (SD = 1.9) and the wild-type JR667 in blue (SD = 0.3). (C) Mapping the causative mutation in *vsc1* by whole genome sequencing of recombinant lines with CB4856. Graphs show the ratio of mapping strain (CB4856) alleles to the total number of reads for 2 different chromosomes. Arrow points to the left arm on chromosome IV that lacks mapping strain polymorphisms. Another chromosome (III) is shown for comparison. Numerical data used for S1 Fig A, B can be found in S2 Data.
(TIF)

S2 Fig The *icb38* mutation represents a new allele of *lin-22* (related to Fig 2). (A-B) PDE neuron number (A) and seam cell number (B) comparison between wild-type animals (*n* = 43) and *lin-22* mutants (*n* = 43). (C-D) Phenotypic comparison between *lin-22* RNAi treated animals (*n* = 30) and control (empty vector) treatment (*n* = 29). RNAi-treated animals show multiple PDE neurons (C) and seam cell number variance (D). (E-F) Phenotypic comparison between *vrp-1* RNAi treated animals (*n* = 35) and control (*n* = 40). No defect was found with regard to number of PDE neurons (E) or seam cell number (F). (G) Quantification of seam cell number in *lin-22(icb38)* mutants based on the *bro-1*CNE∷GFP marker (*n* ≥ 32). (H-I) Phenotypic characterisation of *lin-22(icb49)* in the CB4856 background, showing multiple PDE neurons (*n* ≥ 31) (H) and seam cell number variance (*n* ≥ 30) (I). (J) Quantification of seam cell number in males carrying the *lin-22(icb-38)* mutation (*n* = 31). Note that terminal seam cell number in wild-type males is 18 per lateral side. (K) Heatmap illustrating the relationship between seam cell number counts on 1 lateral side and those on the other lateral side in wild-type and *lin-22(icb38)* animals. The majority of animals show 16 seam cells on both sides in wild-type and moderate correlation of errors (R = 0.37). In *lin-22(icb-38)* mutants, there is even less correlation between the seam cell number deviations on one side and the other (R = 0.23). Black stars show statistically significant changes in the mean with a *t* test or one-way ANOVA and Dunnett’s test; red stars depict changes in variance with a Levene’s median test as follows: *** *P* < 0.001, **** *P* < 0.0001. For PDE scorings, error bars show mean ± SEM and for seam cell number counts error bars show mean ± SD. Numerical data used for S2 Fig A, B, C, D, E, F, G, H, I, J, K can be found in S2 Data. GFP, green fluorescent protein; PDE, post-deirid; SCM, seam cell marker; CNE, conserved non-coding element; RNAi, RNA interference.
(TIF)

S3 Fig *lin-22* promoter conservation and *lin-22* expression analysis (related to Fig 3). (A) Vista analysis (70% identity and 100 base-sliding window) depicting 2 regions (CR1 and CR2) in *lin-22* promoters that are conserved between the following *Caenorhabditis* species: *C*. *elegans*, *C*. *briggsae*, *C*. *remanei*, and *C*. *angaria*. The position of these elements is shown with the *C*. *elegans lin-22* promoter as a reference. Note that CR1 overlaps with Y54G2A.67 that is annotated on Wormbase as a putative noncoding RNA. Part of the CR1 sequence with 2 putative GATA sites and the position of the *icb38* and *ot269* mutations are also shown. (B) *lin-22* smFISH in late L1 wild-type and *lin-22(icb38)* animals. In wild-type *lin-22*, spots were observed in anterior seam cells and not posterior (dashed line marks the seam cell boundary). (C) Quantification of *lin-22* spots in the 4 V1-V4 daughter cells early (*n* ≥ 11) and late (*n* ≥ 22) after the asymmetric division. (D) *lin-22* smFISH in wild-type and *lin-22(icb38)* L4 animals. Note expression in intestinal cells in the mutant (arrows). Nuclei DAPI staining is shown in magenta. (E) Quantification of *lin-22* spots in pooled posterior V1–V4 daughter cells at the L2 asymmetric division stage in wild-type animals treated with control bacteria (*n* = 93), and *elt-1* (n≥57) or *egl-18* RNAi (*n* = 90). Black stars show statistically significant changes in the mean with one-way ANOVA and Dunnett’s test as follows: *** *P* < 0.001, **** *P* < 0.0001. Scale bar in B, D is 10 μm and black spots correspond to mRNAs. Error bars in C, E show mean ± SEM. Numerical data used for S3 Fig C, E can be found in S2 Data. CR1, conserved region 1; CR2, conserved region 2; L1, first larval stage; L2, second larval stage; L4, fourth larval stage; smFISH, single molecule fluorescent in situ hybridization.
(TIF)

S4 Fig Postembryonic seam cell lineage analysis in *lin-22* mutants (related to Fig 4). (A) Heat map illustrating the increase (in blue) or decrease (in red) in cell number output per cell lineage (H0–V6) compared to the wild-type. Each line is an independent lateral side of one animal, while white colour indicates a wild-type cell number output. Note the presence of lineages producing extra and fewer cells within the same lateral side. (B) Seven representative postembryonic lineages of H0–V6 seam cells from *lin-22(icb38)* animals. Solid black lines indicate seam cell fate, gray lines indicate daughters that differentiate into hypodermal cells, and blue dots depict lineages that give rise to PDE neurons. Errors in the lineages that result in terminal seam cell number reduction derive from loss of the L2 symmetric division of V1–V4 cells and adoption of a V5-like pattern (highlighted in red boxes). Hybrid lineages where ectopic neurogenesis co-occurs with seam cell fate maintenance (thus do not change seam cell number) are shown in yellow boxes. Errors that increase the terminal seam cell number, such as V1–V4 symmetric divisions at the L3/L4 stage or H2 symmetric divisions at the L2 stage, are shown in blue and green boxes, respectively. Errors that do not change the terminal seam cell number, such as V cell polarity defects mostly at the L4, V1–V4 cells skipping an asymmetric division, or V1–V4 cells showing an extra asymmetric division are highlighted in gray. Note that timing of divisions is not generally affected to suggest broad developmental timing defects. However, within single lineages we observed a rare manifestation at the L3 stage of a repeat of the L2 pattern of V1–V4 symmetric division followed by an asymmetric division (see lineage right underneath panel A). Asterisks depict lineages that show errors that both decrease and increase the total seam cell number for that lineage. Numerical data used for S4 Fig A can be found in S2 Data. L2, second larval stage; L3, third larval stage; L4, fourth larval stage; PDE, post-deirid.
(TIF)

S5 Fig Gene expression changes associated with gain and loss of cell fate symmetry in *lin-22* mutants (related to Figs 5 and 6). (A) Quantification of *mab-5* expression by smFISH. Note that posterior V(1–4).pp cells express *mab-5* at similar levels to that of wild-type V5. Error bars show mean ± SEM. (B) Quantification of the frequency of symmetrization of *elt-1* expression in the H2 daughters (*n* = 13) and loss of expression in anterior V cell daughters (*n* ≥ 30) in *lin-22(icb38)* mutants assessed by smFISH. (C) Quantification of the frequency of detection of *mab-5* expression in posterior daughters of the H2 (*n* = 10) and V1–V4 (*n* ≥ 20) cells in *lin-22(icb38)* mutants assessed by smFISH. (D) Representative smFISH image of *lin-22(icb38)* mutants using an *elt-1* or *mab-5* probe during the symmetric division of L2 stage and an *egl-18* probe at the asymmetric L2 stage. Arrowheads mark the pattern changes described in this work. Note cell-to-cell variability as V3 in these particular images does not show the described pattern change while adjacent cells do. (E) Quantification of cases in which the V.paa daughter cell in *lin-22(icb-38)* mutants expresses *egl-18* (assessed by smFISH) outside the WT range. Red box marks such cases, grey depicts expression within the WT range, and white when expression was nondetermined due to lack of expression data for this cell. Lines correspond to different animals. Note cell-to-cell variability in the vast majority of animals. (F) Fluorescent images of the wild-type and *lin-22(icb38)* head region with seam cells marked by *scm*∷*GFP*. Note the presence of H cell clusters in *lin-22(icb38)* mutants in which H cells appear to be in duplicates (arrowheads). (G) The occurrence of the above phenotype was scored in early adult animals selected for presence (“positive”; *n* = 17) or absence (“negative”; *n* = 15) of POPHHOP marker expression in the head during the L2 stage. A random population (*n* = 38) was also scored in parallel for the phenotype of interest. Note the significant increase in the frequency of the phenotype in animals selected for the presence of POPHHOP signal (positive) in comparison to both the negative and random populations. (H) *lin-17* smFISH images at the L3 stage showing the entire anterior-posterior axis of a wild-type (same with Fig 6D) and *lin-22(icb38)* mutant animal. Black stars show statistically significant changes with a Fisher’s test: * *P* < 0.05, ** *P* < 0.01. Scale bar in D, F, H is 10 μm, and black spots in D, H correspond to mRNAs. Black bars in B, C, G show frequency ± standard error of the proportion. Numerical data used for S5 Fig A, B, C, G, E can be found in S2 Data. L2, second larval stage; POPHHOP, POP-1 and HMG-helper optimal promoter; smFISH, single molecule fluorescent in situ hybridization.
(TIF)

S6 Fig Gene expression analysis in *lin-22* mutants (related to Figs 5 and 6 and S1 Data). (A) Heat map showing the expression of the top 50 upregulated genes that are statistically significant at FDR-adjusted *P*-value of < 0.1 in *lin-22(icb38)* mutants. (B) Heat map showing the expression of selected genes. Changes in *ceh-16*, *lin-14*, *nhr-25*, *egl-18* in *lin-22(icb38)*, and *lin-22(ot269)* are statistically significant. In both panels, the color of each cell in the heat map indicates the difference in the mean expression level in *lin-22* mutants relative to the wild-type JR667 strain as per the color key.
(TIF)

S7 Fig Developmental defects in other tissues in *lin-22* mutants (related to Fig 7). (A) Quantification of the average number of induced vulval cells in wild-type and *lin-22(icb38)* animals at the early L3 stage (as inferred by scoring at the L4 stage, *n* = 50). (B) Quantification of the number of π cells in wild-type (*n* = 29) and *lin-22(icb38)* (*n* = 34) animals at the L3 stage. (C) Quantification of the number of intestinal nuclei in wild-type and *lin-22(icb38)* animals at the early L1 stage (*n* = 39). Black stars show statistically significant changes in the mean with a *t* test as follows: ** *P* < 0.01. Error bars show mean ± SD. Numerical data used for S7 Fig A, B, C can be found in S2 Data. L1, first larval stage; L3, third larval stage; L4, fourth larval stage.
(TIF)

## Abbreviations

bHLH: basic helix-loop-helix
CR1: conserved region 1
CR2: conserved region 2
CRISPR/Cas9: Clustered Regularly Interspaced Short Palindromic Repeats/CRISPR-associated protein-9
DIC: differential interference contrast
GFP: green fluorescent protein
GWAS: genome-wide association study
Hes: Hairy/Enhancer of Split
L1: first larval stage
L2: second larval stage
L3: third larval stage
L4: fourth larval stage
Lsc: less seam cells phenotype
Msc: more seam cells phenotype
PDE: post-deirid
POPHHOP: POP-1 and HMG-helper optimal promoter
QTL: quantitative trait loci
RNAi: RNA interference
smFISH: single molecule fluorescent in situ hybridization
TCF/LEF: T-cell factor/lymphoid enhancer factor
Vsc: variable seam cell number phenotype
WT: wild-type
