# Supplementary material for: Stochastic loss and gain of symmetric divisions in the C. elegans epidermis perturbs robustness of stem cell number
Source: PLoS Biol. 2017 Nov 6;15(11):e2002429. doi: 10.1371/journal.pbio.2002429 (PMC5690688; doi:10.1371/journal.pbio.2002429)
Supplement: S1 Text — CR1, conserved region 1; CR2, conserved region 2. (DOCX) [file pbio.2002429.s001.docx]

***> lin-22(icb38)***

*Y54G2A.3 exons | mca-3 | lin-22 exons*

CACCGAACCCTACTATTTGTACCTTTTGCGGCGGTTTTGCACTAAAACATGAAACCCATAAATCACTTTCTCTCAAATCAAACGTAAATTGAATTGCTACAAGAGAAGAGGATGAGGTGTGGAACAAGCCGGATATTCCCAAATGTACCAATTATAGGGGGAAAAACATTGAATGTATCTTGAACTTCACCGTCAGTGGGGAAATTGCTTTAAAACATGCCTATCAGGGTTGTGCGGCAAATTTGCCGAATTTGCCGAATTTGCCGAATTTGCCGTTTGCCGAGCTCGGCAAATTTGCCGAATTTGCCGCACACCCCTGATGCCTATGGTACCCCAATGACCTAAAAGCATGGTGAAAAATTTTTAAAAAATTTCCCAGATTTTATATGATTTTTTGAAAACTGAAAAAATCTCAGTTTTTGCCAAATTCCTATTTGAATTTCCCACGTATTTGTGCGTATTTGTCCAGTGGAGCGCACTTGCATCTTTTCAGAAAAAATGATTTTGGAGTGTTTTTTCCCAGTGTTTTTTCAATGTATTTATCCAAAATTATTGAAATCTTGAAAAAATAATATCAAAACTCGAAAAATAATGGAGAAAACACTCCAAAATCATTTTTTCTGAAAAGATGCAAGGGCGCTCCACTGGACAAATACGCACAAATACGCGGGAAATTCAAACAGGAATTTGGCAGAACTGAGATTTTTTCAGTTTTCAAAAAATCATATAAAATCAGGAAATTTTTTAAAAATTTTTTCACCATGATTTTCGGTCATTGTGGTACCATAGGCATGTTTTAAAGCAATTTCCCCATTGGCGCTACTCCTCCTTTAAAAATACCTTTTATTAACGAGGGACGAGAAAAAAAACGGTTTCTAGATCATGGCCGAGGATCCGACCCCATATGTAAAAAAAATTTAGTTATCAGTTTTGATAAGAAAAAAACGGGAAAAATCGGTGAAAAACAAAAGAAAACAAAGCAAGATCTAATTAAGTTGCCGCTAAATCTTGTCACGGGGTTCTGGCCTTCCTCATTGAATTTTTCGCGCTCCATTGACAATCGCCCGTGTACTCCACACGGACAAACACATTTAGTTTTACAACTAGAACCGAGCCGCGACGCGACACGCAACGCGCCGTAAATCTACCCCAGATATGGCCGAGCCAAAATGGCCTATTTCGGCAAACTCTTCCATTTCAATTTATTAGGGATGCCAGAAATCCGTGTCTTATCGGATGTTTGGCCATGGCCTAAAAACCGTGAACTTTGCAATTCTTCGAATGACGTGGCACTTTTGACACCTGTCACCTGATGACCGATAAGAAGTTCTGAAATTTCAAAAAAAAAAAAGAAAATGTTCAGGCTGACAGCAGGTGTCACTGATAAGCTGATAAGCGGTTCGTGTCGAAACTCTCTCACCCCACCTCGCTGTCCCAAAACCATGCATTTTTTGTATTCGCACCCCATAATTGCATAATGATCAACGAGGAACAGCTGCAGCAGCAGCAGCAGCACAGAAGAAAGAAAAAACCAGCGGATGAGGTGACTCAGAAGATGCCGAGTGGTGAAAAGGAGCCGATGGATCCGTACAAAGTGAGTTTTGCGAAAAAAAAATTCTGCCAGAACAAAAAATCCGGCGGATATTTAAACTTATAACTGCGACCAATCAGCGATTCGCTCAGCCCACTTTTCGACCAATCAGCAAAAGTTTGCAGAGTTCAAAGGCGCTGATTGGTTTGAAAGTGAGTGGGGCTTAGCGCTGATTGGTTTCGCATTTTTCGTTTTCTATGACATTGAAACAAGGAGATTTTGTGTCGGAGTGAAGGTGTTACAACGGTAGAGATACTGTAGAGGTATAAGGTACTGTAGGATTACTGTAGTTTCGGAAATTTTGGGTTTCTAGCTTTTGAAGAGGTATTATGTTGGTAGTGGTGGGGGAATAATGTCGAGCTATTGTAGTGGTACTGTAGGGGTACTGTAGGCTTACTGTAGATGTACTGTAGGGATGCTGAGATTTAGGCTTAACTTTAGGCTTAGGCTAAGGCATAGGCTTAGGCTTAGGCTCAGCTTTAGGCTTAGTCCTATGCCTAAGTTTAGGTTCAAGACTAGGCTTAGACTCAAGCTTATTTATTTCGATTTTCAGCTAGGAGCACTGAAAAAAGCACAAAACGAGCGAAAAAACGATTGGATCGCTCTTAGGCTTAGGCTAAGGCTTAGTCTTAGGCTTAGGCTCAGGTTTAGGCTTAGGCTCAAGCTTCTTTATTTCCATTTTCAGCTAGAAGCACTGTAAAAAGCACAAATCGAGCGAAACAACGAATGGATCGCTCTTAGGTTTAGGTTTAGGCCTAGGCTAAGGTTCAGGTTTAGGGTTAGGCTTAGGCTTAGGACTAGGTTTAGGCTCAGGCTTAGGTTTAGGCTTAGGCTTGGGCTTAGGCTTGGGCATAGGCTTAGGCTCAAGCTTATTTATTTCGATTTTCAGCTAGAAGCACTGAAAAAAGCGCAAATCGAGCGAGACAACGAATGGATCGCTTTTAGGCTTAGGTTAAGGCTTAGGCTTAGGTTAAGGCTTAGGCTTAGGCTTAGGACTAGGTTTAGGCTCAAGCTTATCTCTTTCGATTTTCAGCTAGAAGCTCTCAAAAAAGCACAAATCGAGCGAAACAACGAATGGATCGAAAAAATGTCGCCAATCGTGAAATACAAGGTAATTATGAAACACGAGACGTTCCTGTCAAAATTGATATTCCAGATTCAGGAGTACATCCGTAAACAGAGAGCAAAGAAActgaccattttttttagtaatgcatcccaggcctccactgatcatggcaattccacccgaattcctgtcgatttctctgcttttctcgaacttctctctctctctctctctcttttctcactctgttattactttttatttggttaattggttttctttttgactaattttcgatcgatatatcgttgtatatcaatagattttgacatttaaaggcacacgaattaagtcagatgggtctcgacagttctatttttgcgactgcatttttcattgaaattgctctgttttaacgaattttatcgctgtctttacatttttctgttaaaaattgttttaaatcagttttcaacgtacaaaaaaccatttttaaccgaaaaatgtgtagcaaactaccataaccttttttgcgcgccgagacccatctcacagtgaatccgtggaaattccaattcaaaaaaagtgctgccatttcaacaacgttctatcgctatgttctaatccattttcagACTCGAAAGGAGATGAAGCCATGGCTAACGGAAAAGCTGTCGCTGCAGCGGAATCTGACGGAAAGAAGGAACGATCTGTGCTCCAGGCAAAACTTACACGGCTTGCTATTCAAATTGGATATGCTGgtgagtttttgggcgcgaaatttgagctgaaaagtgcaatttttcactgaaaaaaccagaaaattgtttgtgaaatcgattttttgcaacacacatttactgtgaaaaatgcacgctgaaaaaaaaaatttcagaaggaaattgaggctcaagatgagcatcactgctagaaaaaaattttgccagaaccccgaaaaaccgaattttcctatttttttaaaaataacctattttttactaaaatgaccaattttgccaacttttccttgtgtgaacggcaggaatttatatttttttcttcaatttttaccataagttgcagaatttggtattttattatgcgttttttgttgaaaatcctattcagttgatatcgattgaaaacttacctaaatcgacaaaaaacgcgtataaatataaatcaaaattttctatttatgtaaaaattaaataaaagtcaaattccggccgttgtgacaagaaaaaccaaaaaattgaagtttttttgcaaaaattaggacatttttcgcatttttaaattttttttttaattctgagtacctgccttaaaggtagtgtagtccaattttttttgttgctttattagactcaaaattgtctgaaaataccgaatttcataatgaaacttcttgaaaacttctcaaaaaaaaaaagttatgacggctcaaaaaatggcctaaaattagttaaaatttgaaatttgaccgatttatcaagcggctggaaacaatttttcttgaaatcaccgcttcaaagcttgattataaaatgtagttaccttgcgttttcaacttcatttaGGCATTTTAAAGTCGATGGACGGCGAGATTTATTAAACAATATTTAAAATCACGCTGTCCATTTACTATTAAAATACCTGAATGAAGTGGAAAACGCAAGATAACTACATTGTATATTCAAACTTAGACGGTGATTTCAAGAAAAATTGTTTCCAGCCGCTGCGACAAGTTGGTCGAATTTCAAATTTTAACTAATTTTAGGCCATTTTTTGAGCCGTCTTAACGTTTTTTTTGAGAAGTTTTCAAGAAGTTTTATTATGAAATTCGGTGTTTTAAAACAATTTTGAGTTTCATGAAACAATAAAAACTCGACTACACCACCTTTAATTGGGCTTTAGTTGAAACATTCTAGAAATTAAAACCTAAAGCAATTCTGAAAATTACTCAAATATTCCACCTTTTTTTGGAAAATTTAGCAGTTTACCAAAACTTTGACTGAAAATTTTGAAGCATCTATAATTTTTCAAAAGGGTTTTAATAATTTTTTTTCAAATTTCCGCACTGCTTTCTCATCTAGTTTTGAAAATTTCAAAATTTCAATATATGTATTGGTTCACTTTTAACACAGAAAAATCGGATATAGAACTATTTTAATTTGAAATCAACTTAACTTCTAAAAGCTGAAATTTAAAAAAAAAATCGAAAAAAAAATCGAAAAAAGGTCCCGGTATTTTCGGTATTTTATTAGTCCTAAGAACTTTTTTTTTACCACCCAAAAAATGAACAACAAATCACATTTTTGAATGTGTATAATTTTAATCAAAACTACCTAGTGGAACGTTTTTTTTTGTGGTTTTTTGTAATCTCTAGTACTTGGAACTTAATCAGTATTTTCTTGGTTATCACCAGTTTTATTAGTTCTATAGTATATCTATGCAAACTTATACACCTCATGTGGTTTGATTTTTTGAATTGTAATTTTATAAAAGGTTGGCAAAACTCAGAGAGATGTAGACCCATTTTTTTGAAAAAAAAAACACCACAAAATAAAGCCGTTAAATTTTTAATTTTAGACAAAATATTTAAATACAGTGCATTTTTTTCCACTTTAAAGGGGGGGGGGCGCATTTACGCGCGATGGTCTCGACACGCCTCGCACCACCCACTGATTTTTGGGGTGCGTGGCGAGACCAATGGTGTGACCATCGCGCGTAAATGCGCCCCCCTTTAAAGTCGTAGAAGTGGAAAAAATGCACTGTATGGTATATATATATATATATGTATACGTTTACGGCAAATCGGCAAATTGGCAAATTGCCGGAATTATGATTGTCCGGCAAATCGGCAAGCCGGAAAATTGCCGATTTGCTGAATTTGCCGGAAATTTTCGGCAAATTGCGACTTTGCGCATTTTTTTGGAAATTTTAGAACTTTAATTTTAATCGACAAAATTGTACGCATCCTATGAATGTTCCTGCATCTATTTTGTAAAGTAAGCGAATTCCATGAAAATATCTACGGAAAACGGGAAAAAATTTCAAAAAGGCACAGGTTCCAGTGTTTCCGTCTTATATAAAATCCCTCTGAAAAATTCCGGCAAATTGGCAAACCGGCAAAAAAACAAAAATATTTGCCGCCCACCTCCTGATTCAAATGCTTCTCTCTATCGATTTTTCCCTCCAAAGTTTCATGTCAACCGTCCTTTTTCATAAGTTCGGTAGAGAAAGAGAATCGATAAGATCTTGGAGGAGTACTGTAGTTTCTCAATTCTTCATTGAAACAAGTCTTTCTCACCAATCGTGCCGCGTCGCGTGTCTTTACCCCCACTTTTTCCTCTTTCGAAATATAAATTTCAAATTTGATTGACAACAGAAACTTTAAGTTTTATTGGGTTTCTTCATTTACAAGATTTGCCCTATAATACCTATGATACTCAAATTTTGCCGTGCATTCACAAAACGCCTGCCTACGCGCCTACGAGGCATACTCAGATCAAGCCAAACAGTTGTCAACTAGGGCTACTAGGCCTTGGGCTTAGGCTATTCTTTGGACTTGTTGCTTTTTTCGAGCAGTTTTCATGGGCGGACTTTGGAGAATGTCAGTTTTTTTGTTTTTCTGAATTATTTCAGGGGTTCAGCCTAAGCTTAAGCCTAATCCTAAGCCTAAGCCCAAGCCTAAACTAAAAGTAAGCCTAAACCTAAGCCTAATCAAGATAATAAAAGTAAAAAAAACGCGATTCTGCAGAAATCTAACTTGCTCAAAAAAGGTGAAAAGCATAGTCCTCCGCCTAAGCCTACGGCTTAGTCTAAGCTTCAGCCTAAACCTAAGCTTAAGCCTAAGCCCTGTTTGGAGTGTCTCATTTTTCGTTCTATTTTCCTTTTTTGAATTTTTGAATTTTCTGGGATTTCTTTTTCTCTTTTCCTCTCTTTCTCAGCCCAAACCTAAGCCTAAGTCCAAGCCTAAGATAAAGCCTAATCCTAAGCTAGGCCGTCAACTAGGCCACCATCAAAGCTTTCTAGGCAGGCAGTTGTATGAGCCTAGTTAATTAAAATAGTTGGAAATTAAATTTGAAAATTAAAAGAGCTGAATAACATAAAGTCAGAAAAACTACCAATTACATAGTTTTACTGTTCAATAACTTATTTGGTGATTCTATTTTTGCTCTTTTTTATATTCATTCACTGTATCTAATACTTTTTTTTTGACGTTACTAATATTATTACAATTATTACAGAAATGACGTCATTCCTGTGCTCCGATACTGAAATTGAATCCGATGGTGGAATCTCCAGATGCAAGAAGATTGTGAGTTGGTTTTTTTCTGTAATTATTCAAACTTTTGTAATTTCACAGCTGTCCATAATTTTGAAAATCGGCTTAATTTAATTTTCAACATTTTCAGAAAAACAAACCTCTAATGGAGAAGAAACGGAGAGCTCGAATAAACAAGTCACTGTCACAACTAAAACAAATTTTGATTCAAGATGAGCATAAGGTATCGATTTTTCTCAATTATTTATACCTCCGAAAAAACAAGTTTATAAAATTGATAATGGAGATTTTCAAATTTTTAAAATTTTAAGACGAAATTTTGGTAAATTGTCAAGTTTTTAAAAAAGTGAACTTTTTTAGCATTGCTTCAGATCAGGGGCCTCGGCAAGTTGCCGGAATTAAAAATTTTCGCCAAATTGTGGTTTTGCACTTCTTTTTGGAAATTTCAGAATTTCAATTTTAATCGGCAAAATTGTACGCATCCTGTGAATCTTGTTACATCTATTTTAAAAAGTAGGCAATTCTATAAAAGAAAATGGGAAAAATATTTCAAAAAGGCACAGTTTTAAGTGTTTCCGTCTTATAAAAAATCCCTCTAAAATTTTCCGGCAAATTTTATATCCAAGTTGCCGGAACTGAAAAGTTCCGGTAAACCGGCAATGTGCCGAAAATGAAAATTTCTGGGAATTTGGCAAACCGGCAATTTGCCGAAAATGAAAATTTCCGAGGAACCGGCAATTTACCGAAAATGAAAATTTCTGGGAAATCGGCAAACCACCAATTTGCCGAATTGCCGAATTTGCCGGAAAAACGGCAATTGCTGAAAAATTTCGGCAAATTGTAGTTTTGCACCTTTTTCTTGGAAATTTCAGAATTTCAATTTTAATCGGCAAAGTTGTACGCATCATATGAATGTTCCTACATTTATTTTGAAAAGATAAGCAAATTCTATTAAAATATCTGAAGAAAACGGGAAAAAATTTAAAAACGGCACAGTTTTAAGTTTTTCCGCCTTATAAAAATTTCTCTAAACACTTGCGGCAAATTGATACCTAACAAATTGCCAAATTGCCGGAATTGAAAATTTCCAGCAAATCGGCAAACCGGCAAATTGGCGGAAATGTAAATTTCCGGCAAGCCGGAAACTTGCCGAATTTTCCGGAGAAACGGCAATTGCTGAAAATTTTCGCCAAATTGTAGTTTTGCACCTTTTTCTTGGAAATTTCAGAATTTCAATTTTAATCGGCAAAATTGTACGCATCCTGTGAATCTTGTTATGTACATCTATTTTTAAAAGTAGGCAATTCTATAAAAATATCTAAAGAAAACGGGAAAAATTTCAAAAGGGCACAGTTTTAAGTGTTTCCGTCTTATAAAAAATCCCTCTAAAAATTTCCGGCAAATTGGTGTTCGGAAAACGGCAAATCGGCAGTTTGCCAAAAATCTAAATTTCTGGCAAATCGGCAAATCGGCAATTTAGTGATTTGCAAAATTTGTCAGCAAAAAAATTTCCCGAACGGCAATTGCCGCCCACAACTGCTTCAGATTCAAAAATTTGGAATAATTAAATACGAACAATTACAACTAGGGCTGTGCAACCGGACGGCCGGTTTTAGAAAAAAAAGCCACTTTTTAGAACTTTGAAAATTTTCCGAGGGGTATGTTGTTTTCACGGCGGCGGACAATTTCCGAGTTTGGCCACTCGCTATACTTCATCATAGAATTTTATAATGACTGGCCAAACTCGGAAATTGTCCGCCGCCGTGAAAACAACATACCCCTCGGAAAATTTTCAAAGTTCTAAAAAGTGGTTTTTTTCTAAAACCGGACGACCGGATGTCCGGTTGCACAGCTCTTATATACTTAGTAGTGTAAAATTAGTAGAAACGTTTTATTCCGTAGGCAGGTTTGAATGAGAAAAATAACATTTTTTAAGTAGCGCGAGAGATGAGAATTCGCGAATAACGTGCAATTGGCAAGTTGTAAAAAAATCAATAATAGCCTCCGCCTCTATTTGGGCTTCTTTTGGGTATTCCATTTTTTATTCTTATTAATTAATGATGTATCAATTCTATTTCAGAATTCCATCCAACATTCCAAATGGGAAAAAGCTGATATTCTCGAAATGGCTGTCGAATACCTCCAACAACTCCGTAGTGCTCAACCATGCTCCTTATCACCTTCAACATCATCCATTTCAACTCCACCAACTCCAAAAGAAGAAATTCGAAATATTAAAGTACCTCTTAATCCAATAGCTTCATTTCTGAACCCAATGATGCAGCAATACGTGGCATATCAGCAGCTGGCTCAGTTATCAATGTACACTCAATTATTTAATAATCCCGCTGGAGTACCACTGAGAGCAGATGCAGGGGTAACTGCTCAGTCACCAGAGCTGGCAGAAAAGTTAAAAATTGAGGATCGAAGTAGAGTGTAGAATTTGGAATTGTTGTCAAGAGCCACCAGTTTTGATGTGAAATTGATAATCTACTAGTTAGCCACCTAAGGTCTATAAAATAGCTTCAGAGGCAGGGTTTCGGCCTAGTTTTGATGATTTTTGAAGTAACGGTGAATTCTGTAAGCTTTTTTTTTGTTTCGAACAAAAAATCCTGGGGAAAAATTTTATCAAAAAAATCAAAAATGTCAATTTTTTGAATTTGAAATTTTTCAAATTTTTCAAATTTTTTTTAGATCAACGCGTTAAAAAAACTTGCGATGAGCTTTAATCAAAAGTTATTCACAATTTTTTATCTCTAAAAACTTTTAATATTCTGTGAAAACTTTTGGCTTTAGACGAAATCTGTTAATTTTTGGGTAACAAAACTAGAAATTACCAAAAAATCTCCAAGGTTTTTTCCAATTTTTGCAGTTTTTCAATCCAAAAAATTTCTGAAATTTAATAAAAGTCATAAACAACTGATTTTGCAGAATTACTTGTTCTGCAACATCTTACTTGTTCTGAAACAAGTGAAAAGCCTAGTCCTTAGCTTAAGCCTAAGCCTAAGCCTGAACGCCAAACTTTAAAATTTAAAAAATAGTTGAGGGGCATTTTGAAAACACCTTAAGCTCCATAACTCGGCGAGTTTTGATGGAATCAATTTGAAACTTCAGACTTAAGCTTCTTGGTGGCCTATACGACTCTGTGTAAAATTTCGCTTTGATCGGCCAACGGGAACCCTTTCAAAAATGAAAAAAACGTTATAAACCTAAGCCTAATCCTACGCCAAAGCTTGGGCTGTAAGCCCAAGCTTAAGCCTACGCCAAAGCCGGAGTCTAAGCCTAAGCCTAAGCATAAGCCTAAACCAAAGCCTGAGCCCAAGCATAAACCTAAACCGAAGCCTGAGCCTAAGCTTAAACTTCCACATTCTGGTGGCTCTTGTCCTACCTTCCTCTTTAATGTTATTCTTTATTTTTTTCGCCCCTCAAAACTCCGTAGA

**>N2 genomic locus showing the *lin-22(icb38)* deletion and the position of two conserved regions within the *lin-22* promoter.**

*Y54G2A.3 lin-22 lin-22(icb38)* deletion deletion boundaries *CR1 CR2*

CACCGAACCCTACTATTTGTACCTTTTGCGGCGGTTTTGCACTAAAACATGAAACCCATAAATCACTTTCTCTCAAATCAAACGTAAATTGAATTGCTACAAGAGAAGAGGATGAGGTGTGGAACAAGCCGGATATTCCCAAATGTACCAATTATAGGGGGAAAAACATTGAATGTATCTTGAACTTCACCGTCAGTGGGGAAATTGCTTTAAAACATGCCTATCAGGGTTGTGCGGCAAATTTGCCGAATTTGCCGAATTTGCCGAATTTGCCGTTTGCCGAGCTCGGCAAATTTGCCGAATTTGCCGCACACCCCTGATGCCTATGGTACCCCAATGACCTAAAAGCATGGTGAAAAATTTTTAAAAAATTTCCCAGATTTTATATGATTTTTTGAAAACTGAAAAAATCTCAGTTTTTGCCAAATTCCTATTTGAATTTCCCACGTATTTGTGCGTATTTGTCCAGTGGAGCGCACTTGCATCTTTTCAGAAAAAATGATTTTGGAGTGTTTTTTCCCAGTGTTTTTTCAATGTATTTATCCAAAATTATTGAAATCTTGAAAAAATAATATCAAAACTCGAAAAATAATGGAGAAAACACTCCAAAATCATTTTTTCTGAAAAGATGCAAGGGCGCTCCACTGGACAAATACGCACAAATACGCGGGAAATTCAAACAGGAATTTGGCAGAACTGAGATTTTTTCAGTTTTCAAAAAATCATATAAAATCAGGAAATTTTTTAAAAATTTTTTCACCATGATTTTCGGTCATTGTGGTACCATAGGCATGTTTTAAAGCAATTTCCCCATTGGCGCTACTCCTCCTTTAAAAATACCTTTTATTAACGAGGGACGAGAAAAAAAACGGTTTCTAGATCATGGCCGAGGATCCGACCCCATATGTAAAAAAAATTTAGTTATCAGTTTTGATAAGAAAAAAACGGGAAAAATCGGTGAAAAACAAAAGAAAACAAAGCAAGATCTAATTAAGTTGCCGCTAAATCTTGTCACGGGGTTCTGGCCTTCCTCATTGAATTTTTCGCGCTCCATTGACAATCGCCCGTGTACTCCACACGGACAAACACATTTAGTTTTACAACTAGAACCGAGCCGCGACGCGACACGCAACGCGCCGTAAATCTACCCCAGATATGGCCGAGCCAAAATGGCCTATTTCGGCAAACTCTTCCATTTCAATTTATTAGGGATGCCAGAAATCCGTGTCTTATCGGATGTTTGGCCATGGCCTAAAAACCGTGAACTTTGCAATTCTTCGAATGACGTGGCACTTTTGACACCTGTCACCTGATGACCGATAAGAAGTTCTGAAATTTCAAAAAAAAAAAAGAAAATGTTCAGGCTGACAGCAGGTGTCACTGATAAGCTGATAAGCGGTTCGTGTCGAAACTCTCTCACCCCACCTCGCTGTCCCAAAACCATGCATTTTTTGTATTCGCACCCCATAATTGCATAATGATCAACGAGGAACAGCTGCAGCAGCAGCAGCAGCACAGAAGAAAGAAAAAACCAGCGGATGAGGTGACTCAGAAGATGCCGAGTGGTGAAAAGGAGCCGATGGATCCGTACAAAGTGAGTTTTGCGAAAAAAAAATTCTGCCAGAACAAAAAATCCGGCGGATATTTAAACTTATAACTGCGACCAATCAGCGATTCGCTCAGCCCACTTTTCGACCAATCAGCAAAAGTTTGCAGAGTTCAAAGGCGCTGATTGGTTTGAAAGTGAGTGGGGCTTAGCGCTGATTGGTTTCGCATTTTTCGTTTTCTATGACATTGAAACAAGGAGATTTTGTGTCGGAGTGAAGGTGTTACAACGGTAGAGATACTGTAGAGGTATAAGGTACTGTAGGATTACTGTAGTTTCGGAAATTTTGGGTTTCTAGCTTTTGAAGAGGTATTATGTTGGTAGTGGTGGGGGAATAATGTCGAGCTATTGTAGTGGTACTGTAGGGGTACTGTAGGCTTACTGTAGATGTACTGTAGGGATGCTGAGATTTAGGCTTAACTTTAGGCTTAGGCTAAGGCATAGGCTTAGGCTTAGGCTCAGCTTTAGGCTTAGTCCTATGCCTAAGTTTAGGTTCAAGACTAGGCTTAGACTCAAGCTTATTTATTTCGATTTTCAGCTAGGAGCACTGAAAAAAGCACAAAACGAGCGAAAAAACGATTGGATCGCTCTTAGGCTTAGGCTAAGGCTTAGTCTTAGGCTTAGGCTCAGGTTTAGGCTTAGGCTCAAGCTTCTTTATTTCCATTTTCAGCTAGAAGCACTGTAAAAAGCACAAATCGAGCGAAACAACGAATGGATCGCTCTTAGGTTTAGGTTTAGGCCTAGGCTAAGGTTCAGGTTTAGGGTTAGGCTTAGGCTTAGGACTAGGTTTAGGCTCAGGCTTAGGTTTAGGCTTAGGCTTGGGCTTAGGCTTGGGCATAGGCTTAGGCTCAAGCTTATTTATTTCGATTTTCAGCTAGAAGCACTGAAAAAAGCGCAAATCGAGCGAGACAACGAATGGATCGCTTTTAGGCTTAGGTTAAGGCTTAGGCTTAGGTTAAGGCTTAGGCTTAGGCTTAGGACTAGGTTTAGGCTCAAGCTTATCTCTTTCGATTTTCAGCTAGAAGCTCTCAAAAAAGCACAAATCGAGCGAAACAACGAATGGATCGAAAAAATGTCGCCAATCGTGAAATACAAGGTAATTATGAAACACGAGACGTTCCTGTCAAAATTGATATTCCAGATTCAGGAGTACATCCGTAAACAGAGAGCAAAGAAAATGCAACGGAGAAAGTTCTCACTGGCATGTGGTCTGAATCATCGGAATGGGAATGGAGAGCCCAGTTCTTCAGTGTCGCTGAGAAGACGTCCGAAAAAGAAGCCGTCCATGAAAGAGCACAGTTCTTCTGCTCCAGTGCTCTCCAGAAACTTGAGTCAACCAGGGGAAAGTGTTTCAGAATCTCGAAAAGAAGCCTCAGCTGTCTCAAATCGAAGAAAATCGGCACCAATTCAACATATGGACATGAGAGATTTGGATGACTTGGAATGAAAAATAACTTCTTTTTTTTTGATAAATTGTAATTACTCTGAAAGAGTCTGAAAATATATTGTACACTATTATTTTTTGGAACTTTAAAAAATGTTCCCAACGTTTCTAACGATTTTTCATTATATATCATGTTTTCTGGAACTCAATAAAGACTTTTTTTTTATTAAACTTTGCAAATACTCATAACCTCAAAGATTTTGTTTTAAAGGTGGAGTGGAGTCAAAAAATTTGCTCTAAATGACAGAATACAGTCCCAATATACCGAATAATGGAAGCTGCGGAGCTTTGTTGCGCGCAAAACAATAAATCACTCCATAATAGTCAGTTTAAACAAATTTCCCCTCAATTTCTCAGCAATTTGTTGTACACCTCTCCAACCGCTCCCCGCCGTTCCATCTATCACCGAACACAATCTCTCGAATACGTGGCGTTCCCCCCCCCGTATGTCCCAAAACAATTTGTATGACCTTTTATGGCAATCATCCACACCTTCTTGTCGTCTCCCCTTCTCTTAATTTTATCTTGATTTACGTGTTCACCTCAACTCGTGTCAACTATCTCTGCGTCTCTAATCCCCTAACACTATTTCCCGGGGGAGATGACAAGTTGCGCTCCCGCTCGGGTTGTCTCTTGTCCAATTTTATCGTCGTTTTCCTGATTTATCGCTTTTGGAGTAGTAACGAATTTTTTTTGCATGGAGTTCCATGTAAAAACATGCATTAATCATTGATATGTCATCAAAACACAAAGAGTAGATTATCTAGAAAATTCAACCGGAGAAACTCAGCCCCTGGCAACGGAAGTTTTGATCAGTTTTCTCTTTTCTCTTTTCACTTTCATAATGGTTGTGAAGTTTGATAAAATCACAAAATATAACAATTTCTGAAAATTTTCCAGCATGTTTGGCTACCCCTGAACGGATTTAAAGCACTTTGTGCATTTTTCACTAAATTCGAAGCTCGTTAAAATTGCTCCAAAAGCGGTTTTTTTGCACGTTTTTCATATTTAAAAAATTCCAAAAAGTTTTTAGAACTCTTGTAAAACGGAAAAAAATCTGCAAAAATTATTTTTCCAGCAATTTTACGAGTACACAAATTTTTAGCTCCGTGAAAGTTTTTTCAAATTGGTTTTTTACAGACTTGAGTAAACATTTTAGCGGTTGTTTTTTTTGCGAATTTTGTTAAAACTTACATCTATGTTGTAACAAAAATTTTCGAATTTTATTTTCAGAAAAAATTAAAAATTGCAACAAAAAAAATCCAGAATTATTGAAATTGATAGTCTACAAAAATCACTCTTGGAGCAATTTTAAAGGTATGAACAGCTCTCAGTGCACAAGGCCAAGATGTGGACGGAGCCTATTTAGTAATCTTCTAATTTTGTGCATCGGATGTTTAAGTACTTTCCGATACTTTTAGCATATTTTTATTGGCAGTACAAAATATATCAGATTTGCTTACCGTATTTCCTCTATTAGTCTTGCAGCCTCTATTAGTCTTGCACCCCTAAAGACCAATCGAAAATTAGTCTTGCAGCCTCTATTAGTATTGCATGCAAGACAAATAGAGGAAATACGGTAATAGTATTGCACACCTATTTTTGCCAGACCAGCAGTATTTTGTGAAAACTTCACCAATTTCGCCATTTTTAGATAAATTAAATTTTAAAGTGTTCAAATAATTGATAAAACAATAGAAAAGCATGTATTTTGTACGATTTAGACTGTTTCAAGTCAAATTTTCGACGTTTAATGGTCAATTTTACAAAGCTATTGGATCTTCGAAAATTAGTCTTGCAGCCTCTAATGGTCTTGCAGTCTCTATTAGTCTTGCACCCCTACGGGTTGATTAAAAATTAGTCTTGCATGCAAGACTAATAGAGAAAATACAGTAATTATTTTAAAAAATGTTTTTTTTTATGATGGAGTTTCAAAGCTTAAAGATACACGGTTTTATATGGATGGCTCTCGCCACGAATAAAAAAATTGCATAATTTTCTAAATTTTCCGGTCTCAGATATCAAAATTGAAATTTCTCAAAATTGCCATTCGAATAGCCTCTATTCAATTTCAGAAACACTTTTCAGAATACTATCGCCAATGGGGAAATTTTCAAGTTCTACCCTAAAATTGGAAGACCCACTAAATTGACCCACAAAACTCAACTGTGCACCTTCAAAATCAAGCATAACCATGTAGATACATAAATACTCTTTGGTAAAATTGACAAATTGAATCTCATTTGATATCCGGAATGTGTCCTATACCCACTGACTGATGACACTAATCGATCGTATTCCTTTTGGCGCCACCCGTCTCCTTTTTGGAAGACCTCCCGCAGTGCCCGCCCTTATACGTCTTGGGTGGTCTTCAGGAGGGTACGGTACCTGAATTTTGATGTATACGCTTTTTTTTGTTGTATTGCATTGAGGTATTATTTTCATGGTGTCATATGTTTTTGAAATTCATACTGTTTTTTTTTAATTTTTCCAAAAACTTTGGCTCCGCGAGCAAGGTGTTTTAATATTTTATATACATATACATATGTGCATTTTTGTTTGAGCAAACGATTTTTTGATGTTTTCAAAAAATTTTAATTTTTAGAACTTGCAATCAAAATGGAAAAATAATTAGTTTTCACGTATGTATTTAAATTTAGTATTTTTAAAATCAACCTTTTTTCTTTGAATTTTATATTTTAAAGGTGGTATAGTCGAAATTTTTGCTCTATCAGACTCAAAATTGTCTGAAAACACCGAATTTCCTAATGAAACTTCTTGAAACTTCTCAAAAAAAAAAGTTATAGGAGCTCAAAAAATGACCTAAAATTAGTTAAAATTTGGAATTTGACCAGTGGCTGGAACTGGAAACTAATTTTCTTTAAATCACCGTCTAGTTTTAACTTGTAATACCAAAATTAGAAGGTGATATAAAAAAAGTTAGTTTCCAGCCACTGGTCAAATTCCAAATAAAATTTAACTAATTTTAGGTCATTTTTTGAGCCGGCATATCTTTTTTTTGAGCAGTTTTCAAGAAGTTTTATTATGAAATTCGGTGTTTTAAAACAATTTTGAGTTTCATGAAACAATAAAAACTCGACTACACCACCTTTAATTGGGCTTTAGTTGAAACATTCTAGAAATTAAAACCTAAAGCAATTCTGAAAATTACTCAAATATTCCACCTTTTTTTGGAAAATTTAGCAGTTTACCAAAACTTTGACTGAAAATTTTGAAGCATCTATAATTTTTCAAAAGGGTTTTAATAATTTTTTTTCAAATTTCCGCACTGCTTTCTCATCTAGTTTTGAAAATTTCAAAATTTCAATATATGTATTGGTTCACTTTTAACACAGAAAAATCGGATATAGAACTATTTTAATTTGAAATCAACTTAACTTCTAAAAGCTGAAATTTAAAAAAAAAATCGAAAAAAAAATCGAAAAAAGGTCCCGGTATTTTCGGTATTTTATTAGTCCTAAGAACTTTTTTTTTACCACCCAAAAAATGAACAACAAATCACATTTTTGAATGTGTATAATTTTAATCAAAACTACCTAGTGGAACGTTTTTTTTTGTGGTTTTTTGTAATCTCTAGTACTTGGAACTTAATCAGTATTTTCTTGGTTATCACCAGTTTTATTAGTTCTATAGTATATCTATGCAAACTTATACACCTCATGTGGTTTGATTTTTTGAATTGTAATTTTATAAAAGGTTGGCAAAACTCAGAGAGATGTAGACCCATTTTTTTGAAAAAAAAAACACCACAAAATAAAGCCGTTAAATTTTTAATTTTAGACAAAATATTTAAATACAGTGCATTTTTTTCCACTTTAAAGGGGGGGGGGCGCATTTACGCGCGATGGTCTCGACACGCCTCGCACCACCCACTGATTTTTGGGGTGCGTGGCGAGACCAATGGTGTGACCATCGCGCGTAAATGCGCCCCCCTTTAAAGTCGTAGAAGTGGAAAAAATGCACTGTATGGTATATATATATATATATGTATACGTTTACGGCAAATCGGCAAATTGGCAAATTGCCGGAATTATGATTGTCCGGCAAATCGGCAAGCCGGAAAATTGCCGATTTGCTGAATTTGCCGGAAATTTTCGGCAAATTGCGACTTTGCGCATTTTTTTGGAAATTTTAGAACTTTAATTTTAATCGACAAAATTGTACGCATCCTATGAATGTTCCTGCATCTATTTTGTAAAGTAAGCGAATTCCATGAAAATATCTACGGAAAACGGGAAAAAATTTCAAAAAGGCACAGGTTCCAGTGTTTCCGTCTTATATAAAATCCCTCTGAAAAATTCCGGCAAATTGGCAAACCGGCAAAAAAACAAAAATATTTGCCGCCCACCTCCTGATTCAAATGCTTCTCTCTATCGATTTTTCCCTCCAAAGTTTCATGTCAACCGTCCTTTTTCATAAGTTCGGTAGAGAAAGAGAATCGATAAGATCTTGGAGGAGTACTGTAGTTTCTCAATTCTTCATTGAAACAAGTCTTTCTCACCAATCGTGCCGCGTCGCGTGTCTTTACCCCCACTTTTTCCTCTTTCGAAATATAAATTTCAAATTTGATTGACAACAGAAACTTTAAGTTTTATTGGGTTTCTTCATTTACAAGATTTGCCCTATAATACCTATGATACTCAAATTTTGCCGTGCATTCACAAAACGCCTGCCTACGCGCCTACGAGGCATACTCAGATCAAGCCAAACAGTTGTCAACTAGGGCTACTAGGCCTTGGGCTTAGGCTATTCTTTGGACTTGTTGCTTTTTTCGAGCAGTTTTCATGGGCGGACTTTGGAGAATGTCAGTTTTTTTGTTTTTCTGAATTATTTCAGGGGTTCAGCCTAAGCTTAAGCCTAATCCTAAGCCTAAGCCCAAGCCTAAACTAAAAGTAAGCCTAAACCTAAGCCTAATCAAGATAATAAAAGTAAAAAAAACGCGATTCTGCAGAAATCTAACTTGCTCAAAAAAGGTGAAAAGCATAGTCCTCCGCCTAAGCCTACGGCTTAGTCTAAGCTTCAGCCTAAACCTAAGCTTAAGCCTAAGCCCTGTTTGGAGTGTCTCATTTTTCGTTCTATTTTCCTTTTTTGAATTTTTGAATTTTCTGGGATTTCTTTTTCTCTTTTCCTCTCTTTCTCAGCCCAAACCTAAGCCTAAGTCCAAGCCTAAGATAAAGCCTAATCCTAAGCTAGGCCGTCAACTAGGCCACCATCAAAGCTTTCTAGGCAGGCAGTTGTATGAGCCTAGTTAATTAAAATAGTTGGAAATTAAATTTGAAAATTAAAAGAGCTGAATAACATAAAGTCAGAAAAACTACCAATTACATAGTTTTACTGTTCAATAACTTATTTGGTGATTCTATTTTTGCTCTTTTTTATATTCATTCACTGTATCTAATACTTTTTTTTTGACGTTACTAATATTATTACAATTATTACAGAAATGACGTCATTCCTGTGCTCCGATACTGAAATTGAATCCGATGGTGGAATCTCCAGATGCAAGAAGATTGTGAGTTGGTTTTTTTCTGTAATTATTCAAACTTTTGTAATTTCACAGCTGTCCATAATTTTGAAAATCGGCTTAATTTAATTTTCAACATTTTCAGAAAAACAAACCTCTAATGGAGAAGAAACGGAGAGCTCGAATAAACAAGTCACTGTCACAACTAAAACAAATTTTGATTCAAGATGAGCATAAGGTATCGATTTTTCTCAATTATTTATACCTCCGAAAAAACAAGTTTATAAAATTGATAATGGAGATTTTCAAATTTTTAAAATTTTAAGACGAAATTTTGGTAAATTGTCAAGTTTTTAAAAAAGTGAACTTTTTTAGCATTGCTTCAGATCAGGGGCCTCGGCAAGTTGCCGGAATTAAAAATTTTCGCCAAATTGTGGTTTTGCACTTCTTTTTGGAAATTTCAGAATTTCAATTTTAATCGGCAAAATTGTACGCATCCTGTGAATCTTGTTACATCTATTTTAAAAAGTAGGCAATTCTATAAAAGAAAATGGGAAAAATATTTCAAAAAGGCACAGTTTTAAGTGTTTCCGTCTTATAAAAAATCCCTCTAAAATTTTCCGGCAAATTTTATATCCAAGTTGCCGGAACTGAAAAGTTCCGGTAAACCGGCAATGTGCCGAAAATGAAAATTTCTGGGAATTTGGCAAACCGGCAATTTGCCGAAAATGAAAATTTCCGAGGAACCGGCAATTTACCGAAAATGAAAATTTCTGGGAAATCGGCAAACCACCAATTTGCCGAATTGCCGAATTTGCCGGAAAAACGGCAATTGCTGAAAAATTTCGGCAAATTGTAGTTTTGCACCTTTTTCTTGGAAATTTCAGAATTTCAATTTTAATCGGCAAAGTTGTACGCATCATATGAATGTTCCTACATTTATTTTGAAAAGATAAGCAAATTCTATTAAAATATCTGAAGAAAACGGGAAAAAATTTAAAAACGGCACAGTTTTAAGTTTTTCCGCCTTATAAAAATTTCTCTAAACACTTGCGGCAAATTGATACCTAACAAATTGCCAAATTGCCGGAATTGAAAATTTCCAGCAAATCGGCAAACCGGCAAATTGGCGGAAATGTAAATTTCCGGCAAGCCGGAAACTTGCCGAATTTTCCGGAGAAACGGCAATTGCTGAAAATTTTCGCCAAATTGTAGTTTTGCACCTTTTTCTTGGAAATTTCAGAATTTCAATTTTAATCGGCAAAATTGTACGCATCCTGTGAATCTTGTTATGTACATCTATTTTTAAAAGTAGGCAATTCTATAAAAATATCTAAAGAAAACGGGAAAAATTTCAAAAGGGCACAGTTTTAAGTGTTTCCGTCTTATAAAAAATCCCTCTAAAAATTTCCGGCAAATTGGTGTTCGGAAAACGGCAAATCGGCAGTTTGCCAAAAATCTAAATTTCTGGCAAATCGGCAAATCGGCAATTTAGTGATTTGCAAAATTTGTCAGCAAAAAAATTTCCCGAACGGCAATTGCCGCCCACAACTGCTTCAGATTCAAAAATTTGGAATAATTAAATACGAACAATTACAACTAGGGCTGTGCAACCGGACGGCCGGTTTTAGAAAAAAAAGCCACTTTTTAGAACTTTGAAAATTTTCCGAGGGGTATGTTGTTTTCACGGCGGCGGACAATTTCCGAGTTTGGCCACTCGCTATACTTCATCATAGAATTTTATAATGACTGGCCAAACTCGGAAATTGTCCGCCGCCGTGAAAACAACATACCCCTCGGAAAATTTTCAAAGTTCTAAAAAGTGGTTTTTTTCTAAAACCGGACGACCGGATGTCCGGTTGCACAGCTCTTATATACTTAGTAGTGTAAAATTAGTAGAAACGTTTTATTCCGTAGGCAGGTTTGAATGAGAAAAATAACATTTTTTAAGTAGCGCGAGAGATGAGAATTCGCGAATAACGTGCAATTGGCAAGTTGTAAAAAAATCAATAATAGCCTCCGCCTCTATTTGGGCTTCTTTTGGGTATTCCATTTTTTATTCTTATTAATTAATGATGTATCAATTCTATTTCAGAATTCCATCCAACATTCCAAATGGGAAAAAGCTGATATTCTCGAAATGGCTGTCGAATACCTCCAACAACTCCGTAGTGCTCAACCATGCTCCTTATCACCTTCAACATCATCCATTTCAACTCCACCAACTCCAAAAGAAGAAATTCGAAATATTAAAGTACCTCTTAATCCAATAGCTTCATTTCTGAACCCAATGATGCAGCAATACGTGGCATATCAGCAGCTGGCTCAGTTATCAATGTACACTCAATTATTTAATAATCCCGCTGGAGTACCACTGAGAGCAGATGCAGGGGTAACTGCTCAGTCACCAGAGCTGGCAGAAAAGTTAAAAATTGAGGATCGAAGTAGAGTGTAGAATTTGGAATTGTTGTCAAGAGCCACCAGTTTTGATGTGAAATTGATAATCTACTAGTTAGCCACCTAAGGTCTATAAAATAGCTTCAGAGGCAGGGTTTCGGCCTAGTTTTGATGATTTTTGAAGTAACGGTGAATTCTGTAAGCTTTTTTTTTGTTTCGAACAAAAAATCCTGGGGAAAAATTTTATCAAAAAAATCAAAAATGTCAATTTTTTGAATTTGAAATTTTTCAAATTTTTCAAATTTTTTTTAGATCAACGCGTTAAAAAAACTTGCGATGAGCTTTAATCAAAAGTTATTCACAATTTTTTATCTCTAAAAACTTTTAATATTCTGTGAAAACTTTTGGCTTTAGACGAAATCTGTTAATTTTTGGGTAACAAAACTAGAAATTACCAAAAAATCTCCAAGGTTTTTTCCAATTTTTGCAGTTTTTCAATCCAAAAAATTTCTGAAATTTAATAAAAGTCATAAACAACTGATTTTGCAGAATTACTTGTTCTGCAACATCTTACTTGTTCTGAAACAAGTGAAAAGCCTAGTCCTTAGCTTAAGCCTAAGCCTAAGCCTGAACGCCAAACTTTAAAATTTAAAAAATAGTTGAGGGGCATTTTGAAAACACCTTAAGCTCCATAACTCGGCGAGTTTTGATGGAATCAATTTGAAACTTCAGACTTAAGCTTCTTGGTGGCCTATACGACTCTGTGTAAAATTTCGCTTTGATCGGCCAACGGGAACCCTTTCAAAAATGAAAAAAACGTTATAAACCTAAGCCTAATCCTACGCCAAAGCTTGGGCTGTAAGCCCAAGCTTAAGCCTACGCCAAAGCCGGAGTCTAAGCCTAAGCCTAAGCATAAGCCTAAACCAAAGCCTGAGCCCAAGCATAAACCTAAACCGAAGCCTGAGCCTAAGCTTAAACTTCCACATTCTGGTGGCTCTTGTCCTACCTTCCTCTTTAATGTTATTCTTTATTTTTTTCGCCCCTCAAAACTCCGTAGA
