# Supplementary figures and images for: Stochastic loss and gain of symmetric divisions in the C. elegans epidermis perturbs robustness of stem cell number
Source: PLoS Biol. 2017 Nov 6;15(11):e2002429. doi: 10.1371/journal.pbio.2002429 (PMC5690688; doi:10.1371/journal.pbio.2002429)

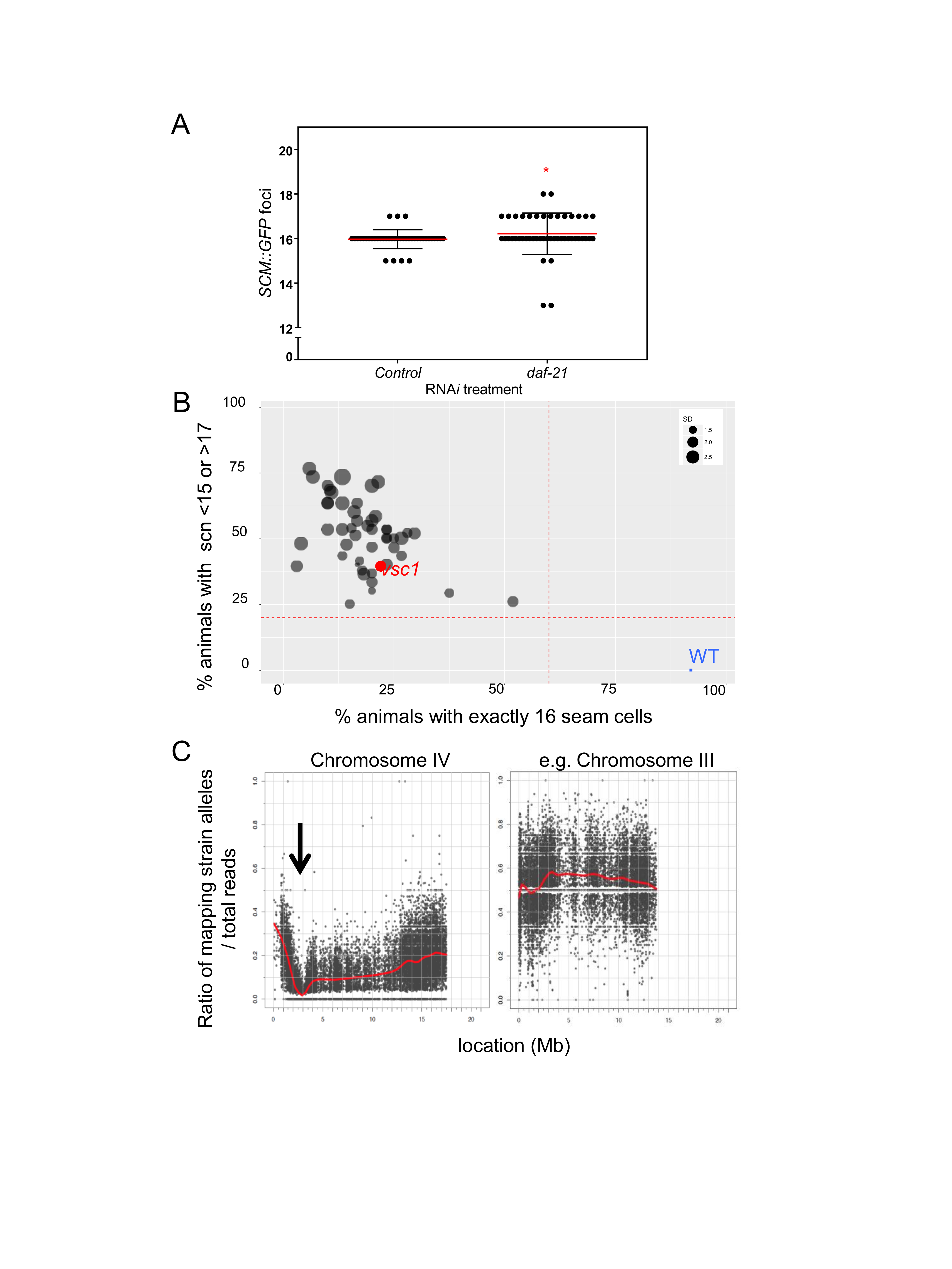

Supplement: S1 Fig — (A) Down-regulation of Hsp90/daf-21 leads to marginal seam cell number variability in the seam (n ≥ 40). Red star depicts change in variance with a Levene’s median test (* P < 0.05). Error bar shows mean ± SD. (B) Graph showing the selected recombinant lines with CB4856 based on quantitative phenotyping of seam cell number standard deviation (SD), percentage of animals with extreme seam cell counts, and percentage of animals with 16 seam cells. Each circle represents 1 line. The parental vsc1 mutant strain is depicted in red (SD = 1.9) and the wild-type JR667 in blue (SD = 0.3). (C) Mapping the causative mutation in vsc1 by whole genome sequencing of recombinant lines with CB4856. Graphs show the ratio of mapping strain (CB4856) alleles to the total number of reads for 2 different chromosomes. Arrow points to the left arm on chromosome IV that lacks mapping strain polymorphisms. Another chromosome (III) is shown for comparison. Numerical data used for S1 Fig A, B can be found in S2 Data. (TIF) [file pbio.2002429.s007.tif]

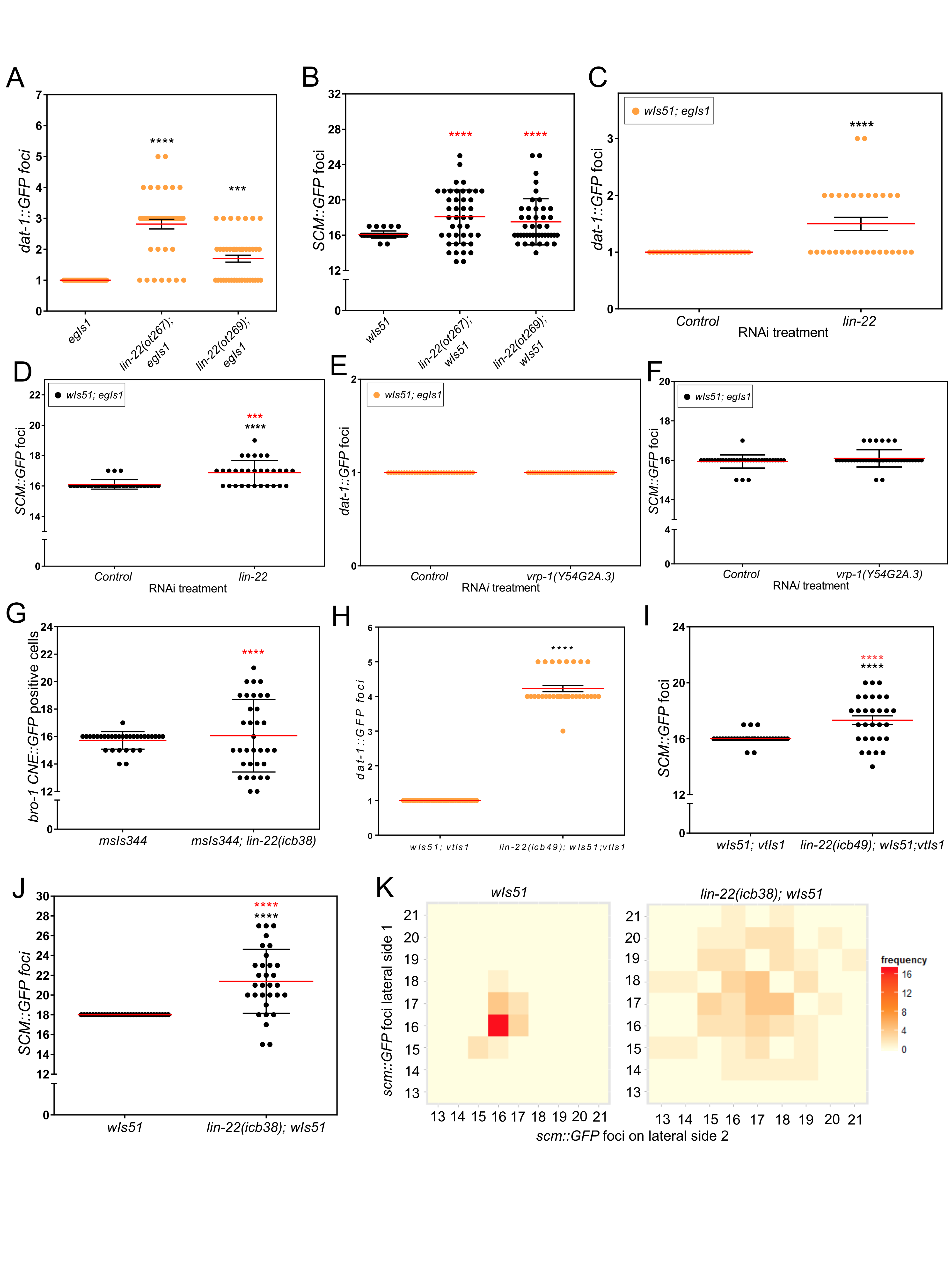

Supplement: S2 Fig — (A-B) PDE neuron number (A) and seam cell number (B) comparison between wild-type animals (n = 43) and lin-22 mutants (n = 43). (C-D) Phenotypic comparison between lin-22 RNAi treated animals (n = 30) and control (empty vector) treatment (n = 29). RNAi-treated animals show multiple PDE neurons (C) and seam cell number variance (D). (E-F) Phenotypic comparison between vrp-1 RNAi treated animals (n = 35) and control (n = 40). No defect was found with regard to number of PDE neurons (E) or seam cell number (F). (G) Quantification of seam cell number in lin-22(icb38) mutants based on the bro-1CNE∷GFP marker (n ≥ 32). (H-I) Phenotypic characterisation of lin-22(icb49) in the CB4856 background, showing multiple PDE neurons (n ≥ 31) (H) and seam cell number variance (n ≥ 30) (I). (J) Quantification of seam cell number in males carrying the lin-22(icb-38) mutation (n = 31). Note that terminal seam cell number in wild-type males is 18 per lateral side. (K) Heatmap illustrating the relationship between seam cell number counts on 1 lateral side and those on the other lateral side in wild-type and lin-22(icb38) animals. The majority of animals show 16 seam cells on both sides in wild-type and moderate correlation of errors (R = 0.37). In lin-22(icb-38) mutants, there is even less correlation between the seam cell number deviations on one side and the other (R = 0.23). Black stars show statistically significant changes in the mean with a t test or one-way ANOVA and Dunnett’s test; red stars depict changes in variance with a Levene’s median test as follows: *** P < 0.001, **** P < 0.0001. For PDE scorings, error bars show mean ± SEM and for seam cell number counts error bars show mean ± SD. Numerical data used for S2 Fig A, B, C, D, E, F, G, H, I, J, K can be found in S2 Data. GFP, green fluorescent protein; PDE, post-deirid; SCM, seam cell marker; CNE, conserved non-coding element; RNAi, RNA interference. (TIF) [file pbio.2002429.s008.tif]

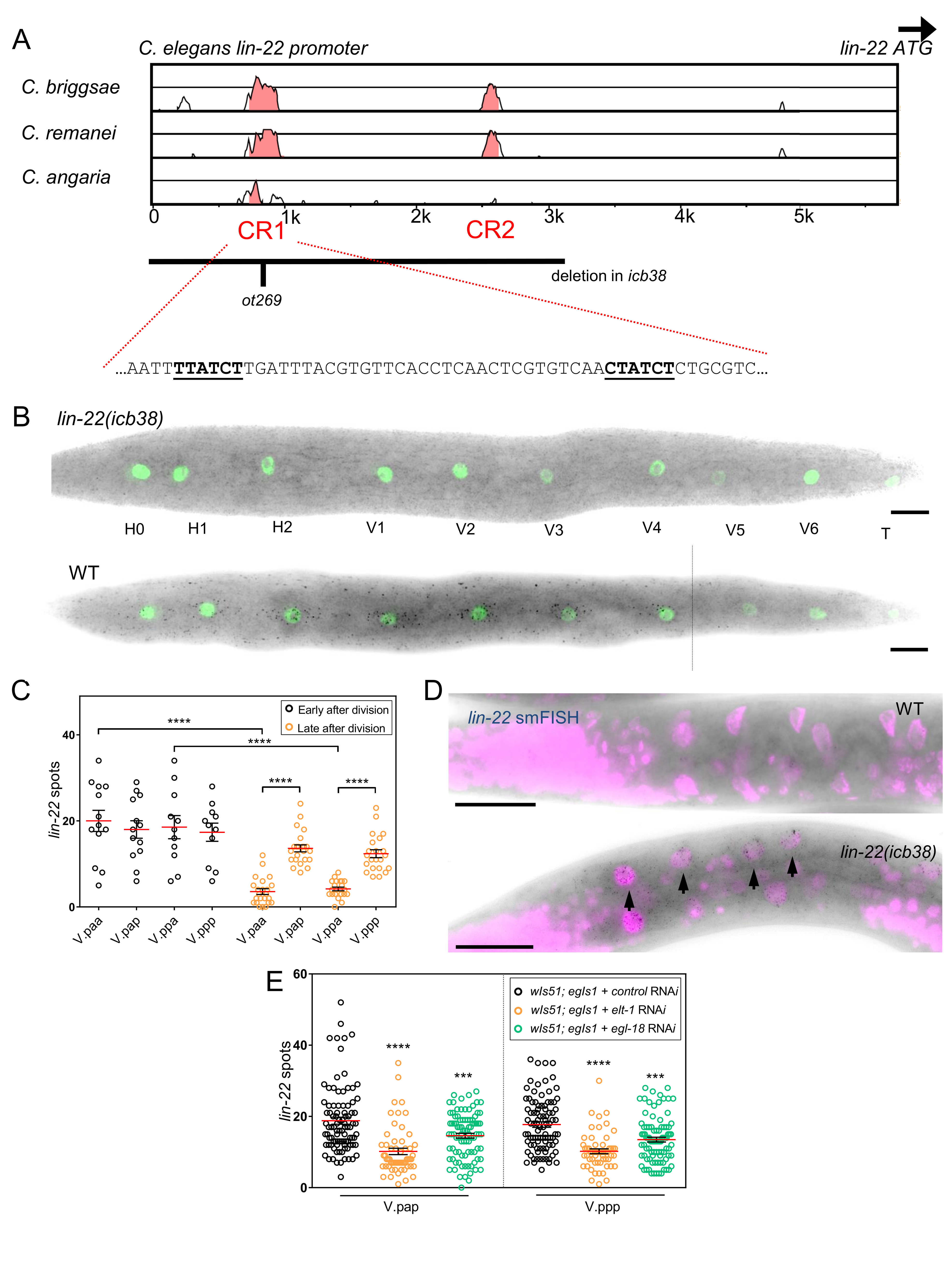

Supplement: S3 Fig — (A) Vista analysis (70% identity and 100 base-sliding window) depicting 2 regions (CR1 and CR2) in lin-22 promoters that are conserved between the following Caenorhabditis species: C. elegans, C. briggsae, C. remanei, and C. angaria. The position of these elements is shown with the C. elegans lin-22 promoter as a reference. Note that CR1 overlaps with Y54G2A.67 that is annotated on Wormbase as a putative noncoding RNA. Part of the CR1 sequence with 2 putative GATA sites and the position of the icb38 and ot269 mutations are also shown. (B) lin-22 smFISH in late L1 wild-type and lin-22(icb38) animals. In wild-type lin-22, spots were observed in anterior seam cells and not posterior (dashed line marks the seam cell boundary). (C) Quantification of lin-22 spots in the 4 V1-V4 daughter cells early (n ≥ 11) and late (n ≥ 22) after the asymmetric division. (D) lin-22 smFISH in wild-type and lin-22(icb38) L4 animals. Note expression in intestinal cells in the mutant (arrows). Nuclei DAPI staining is shown in magenta. (E) Quantification of lin-22 spots in pooled posterior V1–V4 daughter cells at the L2 asymmetric division stage in wild-type animals treated with control bacteria (n = 93), and elt-1 (n≥57) or egl-18 RNAi (n = 90). Black stars show statistically significant changes in the mean with one-way ANOVA and Dunnett’s test as follows: *** P < 0.001, **** P < 0.0001. Scale bar in B, D is 10 μm and black spots correspond to mRNAs. Error bars in C, E show mean ± SEM. Numerical data used for S3 Fig C, E can be found in S2 Data. CR1, conserved region 1; CR2, conserved region 2; L1, first larval stage; L2, second larval stage; L4, fourth larval stage; smFISH, single molecule fluorescent in situ hybridization. (TIF) [file pbio.2002429.s009.tif]

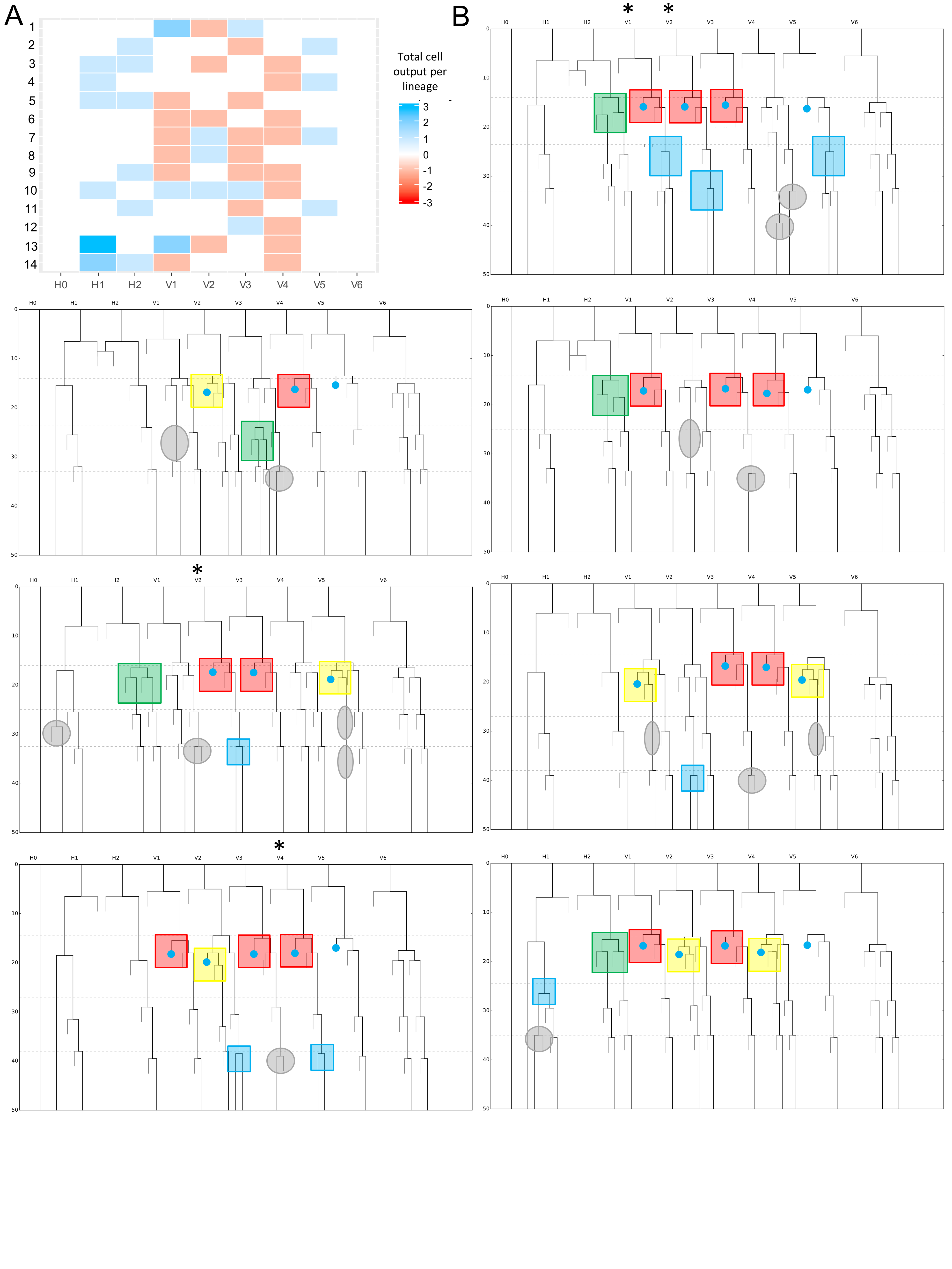

Supplement: S4 Fig — (A) Heat map illustrating the increase (in blue) or decrease (in red) in cell number output per cell lineage (H0–V6) compared to the wild-type. Each line is an independent lateral side of one animal, while white colour indicates a wild-type cell number output. Note the presence of lineages producing extra and fewer cells within the same lateral side. (B) Seven representative postembryonic lineages of H0–V6 seam cells from lin-22(icb38) animals. Solid black lines indicate seam cell fate, gray lines indicate daughters that differentiate into hypodermal cells, and blue dots depict lineages that give rise to PDE neurons. Errors in the lineages that result in terminal seam cell number reduction derive from loss of the L2 symmetric division of V1–V4 cells and adoption of a V5-like pattern (highlighted in red boxes). Hybrid lineages where ectopic neurogenesis co-occurs with seam cell fate maintenance (thus do not change seam cell number) are shown in yellow boxes. Errors that increase the terminal seam cell number, such as V1–V4 symmetric divisions at the L3/L4 stage or H2 symmetric divisions at the L2 stage, are shown in blue and green boxes, respectively. Errors that do not change the terminal seam cell number, such as V cell polarity defects mostly at the L4, V1–V4 cells skipping an asymmetric division, or V1–V4 cells showing an extra asymmetric division are highlighted in gray. Note that timing of divisions is not generally affected to suggest broad developmental timing defects. However, within single lineages we observed a rare manifestation at the L3 stage of a repeat of the L2 pattern of V1–V4 symmetric division followed by an asymmetric division (see lineage right underneath panel A). Asterisks depict lineages that show errors that both decrease and increase the total seam cell number for that lineage. Numerical data used for S4 Fig A can be found in S2 Data. L2, second larval stage; L3, third larval stage; L4, fourth larval stage; PDE, post-deirid. (TIF) [file pbio.2002429.s010.tif]

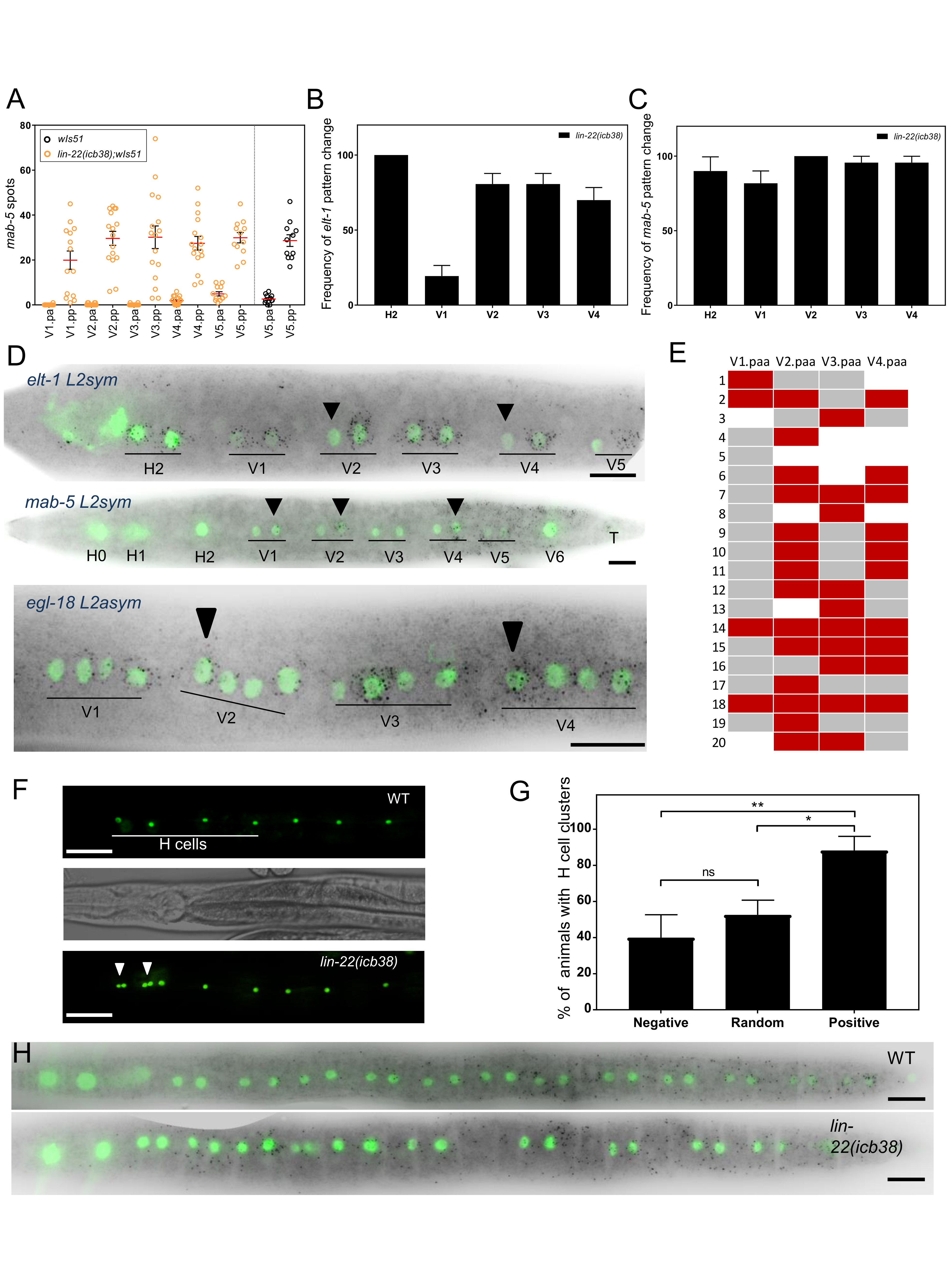

Supplement: S5 Fig — (A) Quantification of mab-5 expression by smFISH. Note that posterior V(1–4).pp cells express mab-5 at similar levels to that of wild-type V5. Error bars show mean ± SEM. (B) Quantification of the frequency of symmetrization of elt-1 expression in the H2 daughters (n = 13) and loss of expression in anterior V cell daughters (n ≥ 30) in lin-22(icb38) mutants assessed by smFISH. (C) Quantification of the frequency of detection of mab-5 expression in posterior daughters of the H2 (n = 10) and V1–V4 (n ≥ 20) cells in lin-22(icb38) mutants assessed by smFISH. (D) Representative smFISH image of lin-22(icb38) mutants using an elt-1 or mab-5 probe during the symmetric division of L2 stage and an egl-18 probe at the asymmetric L2 stage. Arrowheads mark the pattern changes described in this work. Note cell-to-cell variability as V3 in these particular images does not show the described pattern change while adjacent cells do. (E) Quantification of cases in which the V.paa daughter cell in lin-22(icb-38) mutants expresses egl-18 (assessed by smFISH) outside the WT range. Red box marks such cases, grey depicts expression within the WT range, and white when expression was nondetermined due to lack of expression data for this cell. Lines correspond to different animals. Note cell-to-cell variability in the vast majority of animals. (F) Fluorescent images of the wild-type and lin-22(icb38) head region with seam cells marked by scm∷GFP. Note the presence of H cell clusters in lin-22(icb38) mutants in which H cells appear to be in duplicates (arrowheads). (G) The occurrence of the above phenotype was scored in early adult animals selected for presence (“positive”; n = 17) or absence (“negative”; n = 15) of POPHHOP marker expression in the head during the L2 stage. A random population (n = 38) was also scored in parallel for the phenotype of interest. Note the significant increase in the frequency of the phenotype in animals selected for the presence of POPHHOP signal (positive) in co [file pbio.2002429.s011.tif]

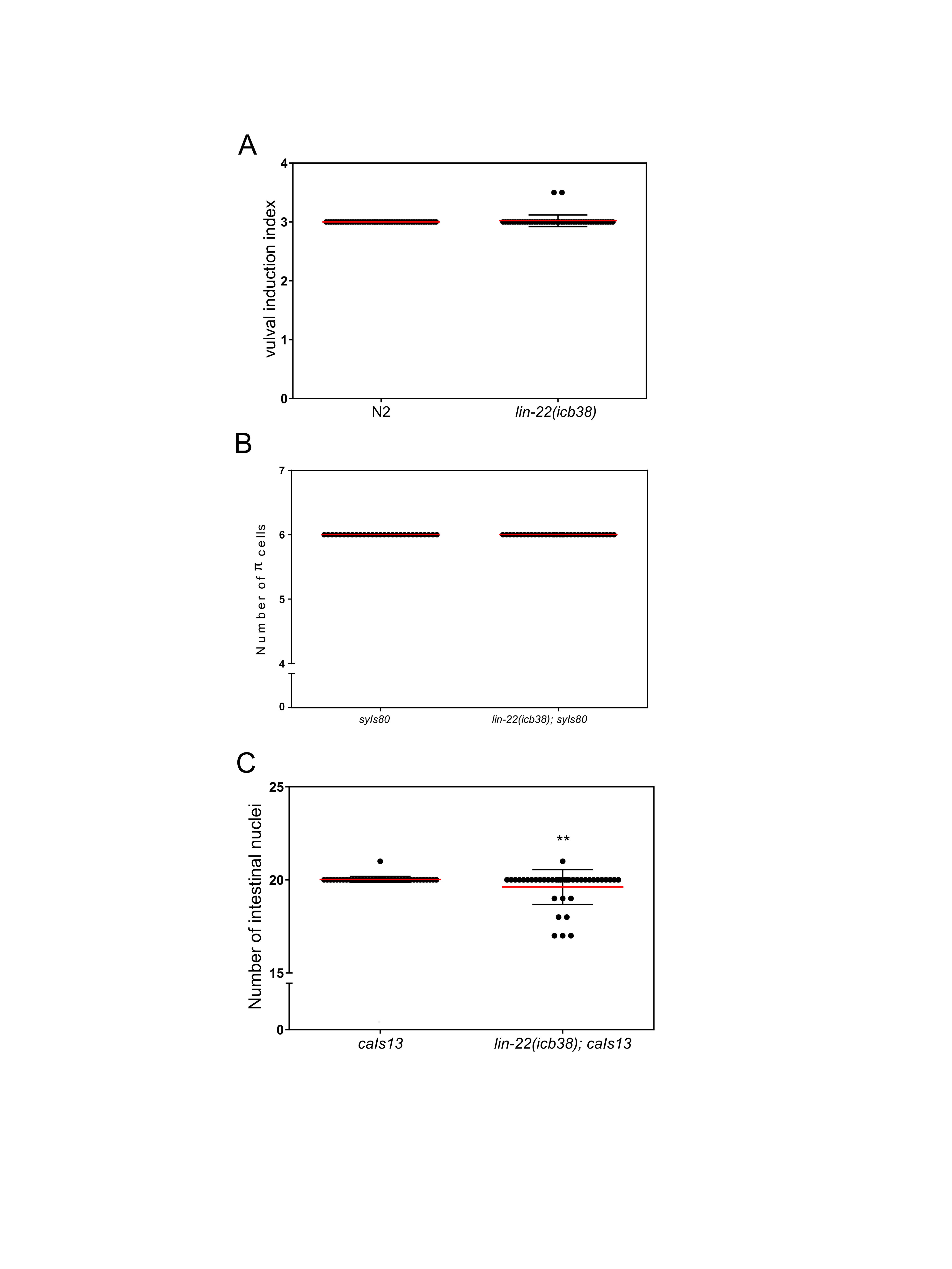

Supplement: S7 Fig — (A) Quantification of the average number of induced vulval cells in wild-type and lin-22(icb38) animals at the early L3 stage (as inferred by scoring at the L4 stage, n = 50). (B) Quantification of the number of π cells in wild-type (n = 29) and lin-22(icb38) (n = 34) animals at the L3 stage. (C) Quantification of the number of intestinal nuclei in wild-type and lin-22(icb38) animals at the early L1 stage (n = 39). Black stars show statistically significant changes in the mean with a t test as follows: ** P < 0.01. Error bars show mean ± SD. Numerical data used for S7 Fig A, B, C can be found in S2 Data. L1, first larval stage; L3, third larval stage; L4, fourth larval stage. (TIF) [file pbio.2002429.s013.tif]
